# Use of Ultrasmall Core-Shell Fluorescent Silica Nanoparticles for Image-Guided Sentinel Lymph Node Biopsy in Head and Neck Melanoma: A Nonrandomized Clinical Trial

**DOI:** 10.1001/jamanetworkopen.2021.1936

**Published:** 2021-03-18

**Authors:** Daniella Karassawa Zanoni, Hilda E. Stambuk, Brian Madajewski, Pablo H. Montero, Danielli Matsuura, Klaus J. Busam, Kai Ma, Melik Z. Turker, Sonia Sequeira, Mithat Gonen, Pat Zanzonico, Ulrich Wiesner, Michelle S. Bradbury, Snehal G. Patel

**Affiliations:** 1 Head and Neck Service, Department of Surgery, Memorial Sloan Kettering Cancer Center, New York, New York; 2 Department of Radiology, Memorial Sloan Kettering Cancer Center, New York, New York; 3 Department of Pathology, Memorial Sloan Kettering Cancer Center, New York, New York; 4 Department of Materials Science and Engineering, Cornell University, Ithaca, New York; 5 Regulatory Oversight and Product Development, Research Technology and Management, Memorial Sloan Kettering Cancer Center, New York, New York; 6 Department of Epidemiology and Biostatistics, Memorial Sloan Kettering Cancer Center, New York, New York; 7 Department of Medical Physics, Memorial Sloan Kettering Cancer Center, New York, New York; 8 Memorial Sloan Kettering–Cornell Center for Translation of Cancer Nanomedicines, Memorial Sloan Kettering Cancer Center, New York, New York; 9 Molecular Pharmacology Program, Memorial Sloan Kettering Cancer Center, New York, New York

## Abstract

**Importance:**

Sentinel lymph node (SLN) mapping agents approved for current surgical practice lack sufficient brightness and target specificity for high-contrast, sensitive nodal visualization.

**Objective:**

To evaluate whether an ultrasmall, molecularly targeted core-shell silica nanoparticle (Cornell prime dots) can safely and reliably identify optically avid SLNs in head and neck melanoma during fluorescence-guided biopsy.

**Design, Setting, and Participants:**

This nonrandomized clinical trial enrolled patients aged 18 years or older with histologically confirmed melanoma in whom SLN mapping was indicated. Exclusion criteria included known pregnancy, breast-feeding, or medical illness unrelated to the tumor. The trial was conducted between February 2015 and March 2018 at Memorial Sloan Kettering Cancer Center, with postoperative follow-up of 2 years. Data analysis was conducted from February 2015 to March 2018.

**Interventions:**

Patients received standard-of-care technetium Tc 99m sulfur colloid followed by a microdose administration of integrin-targeting, dye-encapsulated nanoparticles, surface modified with polyethylene glycol chains and cyclic arginine-glycine–aspartic acid–tyrosine peptides (cRGDY-PEG-Cy5.5-nanoparticles) intradermally.

**Main Outcomes and Measures:**

The primary end points were safety, procedural feasibility, lowest particle dose and volume for maximizing nodal fluorescence signal, and proportion of nodes identified by technetium Tc 99m sulfur colloid that were optically visualized by cRGDY-PEG-Cy5.5-nanoparticles. Secondary end points included proportion of patients in whom the surgical approach or extent of dissection was altered because of nodal visualization.

**Results:**

Of 24 consecutive patients enrolled (median [interquartile range] age, 64 [51-71] years), 18 (75%) were men. In 24 surgical procedures, 40 SLNs were excised. Preoperative localization of SLNs with technetium Tc 99m sulfur colloid was followed by particle dose-escalation studies, yielding optimized doses and volumes of 2 nmol and 0.4 mL, respectively, and maximum SLN signal-to-background ratios of 40. No adverse events were observed. The concordance rate of evaluable SLNs by technetium Tc 99m sulfur colloid and cRGDY-PEG-Cy5.5-nanoparticles was 90% (95% CI, 74%-98%), 5 of which were metastatic. Ultrabright nanoparticle fluorescence enabled high-sensitivity SLN visualization (including difficult-to-access anatomic sites), deep tissue imaging, and, in some instances, detection through intact skin, thereby facilitating intraoperative identification without extensive dissection of adjacent normal tissue or nerves.

**Conclusions and Relevance:**

This study found that nanoparticle-based fluorescence-guided SLN biopsy in head and neck melanoma was feasible and safe. This technology holds promise for improving lymphatic mapping and SLN biopsy procedures, while potentially mitigating procedural risks. This study serves as a first step toward developing new multimodal approaches for perioperative care.

**Trial Registration:**

ClinicalTrials.gov Identifier: NCT02106598

## Key Points

**Question:**

Can the favorable properties of an ultrasmall fluorescent core-shell silica nanoparticle aid real-time image-guided detection, localization, and surgical management of sentinel lymph nodes (SLNs) in patients with head and neck melanoma?

**Findings:**

In this nonrandomized clinical trial of 24 patients, real-time, particle-based fluorescence imaging of SLNs was feasible and safe at the microdosing level and enabled deep-tissue nodal detection. There was high concordance in identifying nodes between preoperative lymphoscintigraphy and particle-based fluorescence-guided biopsy.

**Meaning:**

The findings of this study suggest that ultrabright, optical contrast–conferring particles for SLN identification hold promise for overcoming current probe limitations and improving surgical outcomes.

## Teaser

This nonrandomized clinical trial evaluates whether an ultrasmall, molecularly targeted core-shell silica nanoparticle can safely and reliably identify optically avid SLNs in head and neck melanoma during fluorescence-guided biopsy.

## Introduction

The accurate localization of tumor-draining lymph nodes and nodal micrometastases in sentinel lymph node (SLN) biopsy procedures is crucial to staging and treating melanoma.^1^ However, current surgical practice lacks ultrabright, target-specific imaging probes that can reliably guide real-time intraoperative nodal detection and treatment of metastases.^2,3,4,5^

Since the implementation of SLN biopsy procedures for early-stage melanoma,^6^ advances in the development of molecularly targeted intraoperative imaging probes have been scarce. The few approved SLN mapping agents, such as technetium Tc 99m sulfur colloid, technetium Tc 99m tilmanocept,^7^ and blue dye, lack a strong optical signal to guide real-time intraoperative identification, and they are limited by nonspecific high macrophage uptake and what is known as shine-through if SLNs are close to the primary lesion.^5^ Lymphoscintigraphy with technetium Tc 99m is also subject to poor spatial resolution (approximately 10 mm) and, therefore, imprecise SLN localization.^8^ Collectively, these considerations highlight a critical need to advance the adoption of new optical imaging probes that generate high-contrast, reliably localized SLNs and identify nodal metastases while potentially enabling real-time tailoring of surgical approach and reducing the extent of surgical dissection, duration of anesthesia time, and risk of nerve injury.

To address these issues, we developed and clinically translated a first-in-kind ultrasmall (sub–8-nm diameter) integrin-targeting, ultrabright, fluorescent core-shell silica nanoparticles (Cornell prime dots).^9,10,11,12,13^ The superior brightness and photostability of fluorescent nanoparticles relative to free dyes is the result of encapsulating near-infrared (NIR) dyes (ie, Cy5.5) within its silica core matrix.^14,15,16^ In addition, as α_ν_ integrins are known to be highly expressed on the surface of neoangiogenic endothelial and melanoma cells, we functionalized the particle surface with multiple integrin-targeting peptides (ie, cyclic arginine-glycine–aspartic acid–tyrosine [cRGD]) via polyethylene glycol chains (PEGs) to create cRGDY-PEG-Cy5.5-nanoparticles.^10,11,17^ Relative to native monovalent peptides, the multivalent nanoparticle platform shows enhanced cellular binding and internalization.^18^

In this study, we present the results of a phase 1/2a particle-driven SLN mapping trial in patients with head and neck melanoma, an orphan disease, using cRGDY-PEG-Cy5.5-nanoparticles and a handheld NIR fluorescence camera system (Spectrum [Quest Medical Imaging]) for accurate intraoperative detection and high-contrast visualization of SLNs, which had been localized preoperatively with standard-of-care technetium Tc 99m sulfur colloid. The particle probe described is, to the best of our knowledge, the first inorganic renally clearable and optically active platform in the United States to have reached clinical trials in patients with melanoma. Primary end points aimed to assess particle safety, procedural feasibility, the particle dose and volume that maximize nodal fluorescence signal and image contrast, and the proportion of nodes identified by technetium Tc 99m sulfur colloid that were optically visualized by cRGDY-PEG-Cy5.5-nanoparticles (ie, concordance). Secondary study end points included time from injection to SLN visualization, the proportion of patients in whom the surgical approach or extent of dissection was subjectively altered, risk of reduced nerve injury, and shortened duration of anesthesia because of improved SLN visualization. The distinct optical and structural properties of nanoparticles suggested that key limitations of technetium Tc 99m sulfur colloid could potentially be overcome and that the proportion of excised SLNs identified by technetium Tc 99m sulfur colloid may be concordant with that visualized by nanoparticles.

## Methods

### Study Design and Patient Population

This clinical trial was approved by the institutional review board at the Memorial Sloan Kettering Cancer Center (MSKCC) and cleared by the US Food and Drug Administration, with physician-sponsored Investigational New Drug 121544. The trial protocol appears in Supplement 1. All referred patients provided informed written consent to proceed with standard-of-care technetium Tc 99m sulfur colloid lymphoscintigraphy (eAppendix in Supplement 2) and intraoperative NIR fluorescence imaging using cRGDY-PEG-Cy5.5-nanoparticles. Particles were administered in the surgical suite under anesthesia. This self-controlled, nonrandomized clinical trial followed the Transparent Reporting of Evaluations With Nonrandomized Designs (TREND) reporting guideline.

Eligibility criteria included being aged 18 years or older with a histologically confirmed diagnosis of cutaneous melanoma in which SLN mapping was indicated.^19^ Participants were clinically node-negative by history, physical examination, and ultrasonographic evaluation.^20^ Patient exclusion criteria included known pregnancy, breast-feeding, or uncontrolled intercurrent medical illness unrelated to the tumor which, in the opinion of the attending staff, contraindicated administering the study agent. All patients were followed up during a 2-year period.

Given that this was a pilot trial to determine whether the preclinical experience and observations would translate to the operating room, sample size was not chosen based on power calculations but rather on estimating concordance between technetium Tc 99m sulfur colloid and cRGDY-PEG-Cy5.5-nanoparticles. With 31 patients, the expected half-length of a 95% CI, assuming 90% concordance, was 12%.

### Fluorescence Image-Guided SLN Biopsy Using cRGDY-PEG-Cy5.5-Nanoparticles

#### Particle Synthesis and Characterization

Ultrasmall, fluorescent core-shell silica nanoparticles (cRGDY-PEG-Cy5.5-nanoparticles) were produced using a previously described 1-pot synthesis approach (eAppendix in Supplement 2).^10,11^ They were purified using gel permeation chromatography, sterile processed, and characterized by fluorescence correlation spectroscopy and UV-visible spectroscopy (eTable 1 in Supplement 2).^11,17^

#### Dosing Strategy

Preoperative localization of SLNs was performed with technetium Tc 99m sulfur colloid (eAppendix in Supplement 2); this was followed by particle dose-escalation studies. cRGDY-PEG-Cy5.5-nanoparticles were intradermally injected into intact skin at the microdosing level (<30 nmol) using a 4-quadrant approach around the primary tumor site and predefined dose-escalation scheme (eFigure 1 in Supplement 2) to determine the minimum dose and volume needed to achieve the primary end points. Initial dosing estimates were based on our experience in both small- and large-animal melanoma models and suggested a safe total starting dose in humans of approximately 1.2 nmol/dose for distribution. These parameters, as well as the time from injection to SLN visualization (phase 2a study), were adjusted and recorded for each patient. As much as 1 mL and 12 nmol of cRGDY-PEG-Cy5.5-nanoparticles were injected (eFigure 1 in Supplement 2). Vital signs were monitored before and after particle administration.

#### Real-Time Intraoperative NIR Fluorescence Imaging

Real-time NIR optical scanning of the primary tumor site and SLNs within the surgically exposed nodal basin was performed using a handheld Spectrum fluorescence camera system (Quest Medical Imaging) (eAppendix in Supplement 2). The Cy5.5 mode used excitation and emission wavelengths of 680 nm and 710 nm, respectively, and the fluorescence images were superimposed on simultaneously acquired color images of the surgical field. Image streams were recorded using the Spectrum Capture Suite from the start of injection to biopsy and ex vivo imaging to document lymphatic flow and accumulation of cRGDY-PEG-Cy5.5-nanoparticles in SLNs. Nodes exhibiting a signal-to-background ratio (SBR) of 1.1 or greater in situ were considered positive by NIR fluorescence (eAppendix in Supplement 2). Qualitative comparisons were made between technetium Tc 99m sulfur colloid and nodal fluorescence signal prior to and after excising the nodes in question for pathologic evaluation.

### Statistical Analysis

Safety was evaluated in terms of vital signs and adverse events (ie, inpatient hospitalization or prolongation of existing hospitalization, persistent or significant incapacity, substantial disruption of the ability to conduct normal life functions, or mortality). A primary efficacy end point was the patient concordance rate, ie, the proportion of nodes successfully identified by preoperative technetium Tc 99m sulfur colloid that were optically visualized by cRGDY-PEG-Cy5.5-nanoparticles. The proportion of nodes that were detected only by technetium Tc 99m sulfur colloid alone or by cRGDY-PEG-Cy5.5-nanoparticles alone was also evaluated. Concordance between technetium Tc 99m sulfur colloid and cRGDY-PEG-Cy5.5-nanoparticles was evaluated using 95% exact binomial confidence intervals. Statistical analysis was conducted in R version 3.6 (R Project for Statistical Computing). Statistical significance was set at *P* < .05, and all tests were 2-tailed.

## Results

Of 24 consecutive patients (median [interquartile range] age, 64 [51-71] years) enrolled from February 2015 to March 2018, 18 (75%) were men (Table 1). Most patients had stage IB or IIA melanoma, with 7 (29%) designated as stage IA, following National Comprehensive Cancer Network (NCCN) guidelines^19^ (eAppendix in Supplement 2). Data were accrued and tabulated for phase 1 (15 patients [63%]; median dose, 2 nmol; range, 0.6-4.0 nmol) and phase 2a (9 patients [37%]; dose, 2 nmol) melanoma studies (eTable 2 in Supplement 2). Dose-escalation studies led to an optimal particle concentration, dose, and volume of 5 nmol/mL, 2 nmol, and 0.4 mL, respectively, with the optimum values of these parameters corresponding to maximum image contrast (tumor-to-background [TB] ratio) of approximately 40. The timing interval between injection and nodal visualization varied between less than 30 minutes to 80 minutes, with delays longer than 30 minutes reflecting cases in which skin graft placement and/or primary lesion excision occurred prior to SLN mapping. However, no appreciable time-dependent difference in fluorescence signal intensity or sensitivity in detecting SLNs was observed. The very high contrast levels achieved in real-time enabled reliable visualization of in situ and ex vivo SLNs and diseased nodes (Table 2) as well as accurate discrimination of nodes from adjacent fibrofatty soft tissue and peritumoral injection sites. On histopathologic examination, 40 SLNs were assessed; SLNs harvested from 6 nodes in 6 patients harbored metastatic melanoma. Three of these patients (50%) had 1 node each resected, 2 (33%) had 2 nodes resected, and a sixth patient (17%) had 4 nodes excised. There were no false-negative results, ie, there were no pathologically confirmed metastatic nodes that were not visualized with nanoparticles, except in 2 cases in which camera system malfunction precluded assessment. There were no adverse events observed in any of the patients, and there were no reported cases of nerve injury.

**Table 1.** Demographic, Clinical, and Pathologic Characteristics

Characteristic	Patients, No. (%) (N = 24)
Sex	
Male	18 (75)
Female	6 (25)
Age, median (IQR), y	64 (51-71)
Breslow depth, median (IQR), mm	1.4 (0.9-2.3)
Ulceration	
Identified	6 (25)
Not identified	18 (75)
Mitosis	
<1/mm^2^	9 (38)
≥1/mm^2^	15 (62)
SLN removed, median (IQR), No.	2 (1-2)
SLN resected	
Benign, No./total No. (%)	34/40 (85)
Metastatic, No./total No. (%)	6 /40(15)
cT stage	
1a	4 (17)
1b	4 (17)
2a	8 (33)
2b	2 (8)
3a	4 (17)
3b	2 (8)
Clinical stage	
IA	4 (17)
IB	12 (50)
IIA	6 (25)
IIB	2 (8)
pT stage	
1a	4 (17)
1b	3 (13)
2a	7 (29)
2b	2 (8)
3a	5 (21)
3b	1 (4)
4b	2 (8)
pN stage	
0	18 (75)
1a	2 (8)
2c	4 (17)
Pathological stage	
IA	7 (29)
IB	6 (25)
IIA	3 (13)
IIB	1 (4)
IIC	1 (4)
IIIA	1 (4)
IIIB	1 (4)
IIIC	4 (17)

Abbreviations: cT, clinical tumor; IQR, interquartile range; pN, pathological nodal; pT, pathological tumor; SLN, sentinel lymph node.

**Table 2.** Summary of Phase 1/2a Data for All 24 Patients

Measures	Results
Adverse events, No.	0
Nerve injury, No.	0
Cases with improved surgical procedure, No.^a^	4
Optimal concentration	5 nmol/mL
Dose	2 nmol in 0.4 mL
Minimum dose	0.5 nmol in 0.4 mL
Maximum dose	4 nmol in 0.4 mL
Time from dose to first node visualization, range, min	30-80
Maximum signal-to-background ratio	40
Nodes excised for histology, No.	40
Pathology-positive nodes, No.	6
Fluorescent nodes that were also positive for pathology, No./total No.^b^	5/6

^a^ Use of nanoparticles led to subjective assessments indicating altered surgical approach, reduced dissection, identifying small (ie, <4 mm) nodes, and discrimination of node from adjacent primary lesion.

^b^ There was a camera malfunction in 1 case.

Although 40 SLNs underwent histopathological evaluation, 9 nodes were precluded from establishing whether the proportion of lymph nodes identified by technetium Tc 99m sulfur colloid was equivalent to that for cRGDY-PEG-Cy5.5-nanoparticle visualization because of technical factors relating to early dose-optimization efforts (cases 1 and 2) and camera malfunction (cases 11 and 13) (eTable 2 in Supplement 2). In cases 1 and 2, nodal visualization was not achievable at very low doses; these cases were excluded so as not to confound analysis of concordance in cases affected by poorly understood technical factors. In cases 11 and 13, the fluorescence camera system malfunctioned for reasons unknown at the time.

Nonetheless, concordance was established between technetium Tc 99m sulfur colloid and nanoparticles in 28 of 31 (90%; 95% CI, 74%-98%) cases of ex vivo SLNs (Table 3). Patient-level concordance, ie, the proportion of patients in whom at least 1 lymph node was detected by either technetium Tc 99m sulfur colloid or cRGDY-PEG-Cy5.5-nanoparticles, was 100%. In no patients did fluorescence fail to identify at least a single node, with 1 SLN (3%) showing fluorescence, but no radioactivity (eTable 2 in Supplement 2).

**Table 3.** Identification and Status of Ex Vivo SLNs Detected

Probes for SLN detection	Ex vivo SLNs, No. (%)		
	Total (N = 31)	Nonmetastatic (n = 26)	Metastatic (n = 5)
Technetium Tc 99m sulfur colloid and nanoparticles	28 (90)	23 (88)	5 (100)
Only technetium Tc 99m sulfur colloid	2 (6)	2 (8)	0
Only nanoparticles	1 (3)	1 (4)	0

Abbreviation: SLN, sentinel lymph nodes.

eTable 3 in Supplement 2 lists differences in techniques, anesthesia time, sensitivity, and resolution of cRGDY-PEG-Cy5.5-nanoparticles vs traditional technetium Tc 99m sulfur colloid without fluorescence-guided visualization. While a randomized comparison with the traditional paradigm was not the aim of this Phase 1/2a study design, in some patients, the use of targeted nanoparticles did result in modification of the surgical approach and/or a significant reduction in planned surgical dissection for SLN identification. Four patients (17%) underwent shorter surgeries and anesthesia times than they otherwise would have because extensive dissection to expose nodes was not needed; that is, early intraoperative visualization of SLNs made them easier to resect. In 2 patients (8%), nodes were easily visualized through the skin (Video), leading to a less invasive and faster surgery. In other cases, nodes close to the primary lesion (injection site), which precluded their identification by gamma counting, were successfully visualized; these SLNs would likely have been missed without intraoperative optical imaging. On the basis of subjective assessments by the operating surgeon, we estimated approximately 30% to 50% reduction in the duration of the surgery using cRGDY-PEG-Cy5.5-nanoparticles compared with technetium Tc 99m sulfur colloid alone. This range reflected variations in the type of procedure, depth of nodes from the skin surface, and the need for nerve dissection, in addition to other technical factors, such as equipment setup.

**Video.** Fluorescent Silica Nanoparticle–Guided Sentinel Lymph Node Biopsy in Melanoma Real-time intraoperative visualization and dissection of a postauricular sentinel lymph node following local injection of integrin-targeting, dye-encapsulated nanoparticles, surface modified with polyethylene glycol chains and cyclic arginine-glycine–aspartic acid–tyrosine peptides (cRGDY-PEG-Cy5.5-nanoparticles), as an imaging probe around the primary scalp melanoma. See Figure 2 for step-by-step details.

**Figure 1.**
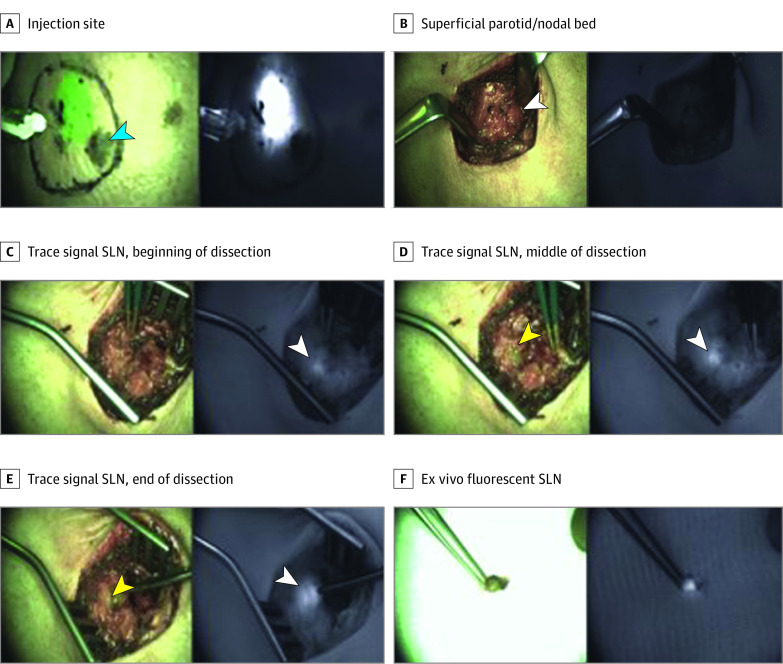
Nanoparticle-Aided Fluorescence Detection of a Metastatic Intraparotid Sentinel Lymph Nodes (SLN) Intradermal injection of a male patient in his 60s with integrin-targeting, dye-encapsulated nanoparticles, surface modified with polyethylene glycol chains and cyclic arginine-glycine–aspartic acid–tyrosine peptides near a scalp melanoma (A), shown in both composite (green signal) and adjacent fluorescence (white signal) displays. Progressive increase in fluorescence signal, seen as a blush within the nodal bed (B-E) on both composite and fluorescence displays, corresponds with an intraparotid SLN approximately 1.5 to 2 cm deep. F, Fluorescence signal within the ex vivo SLN.

**Figure 2.**
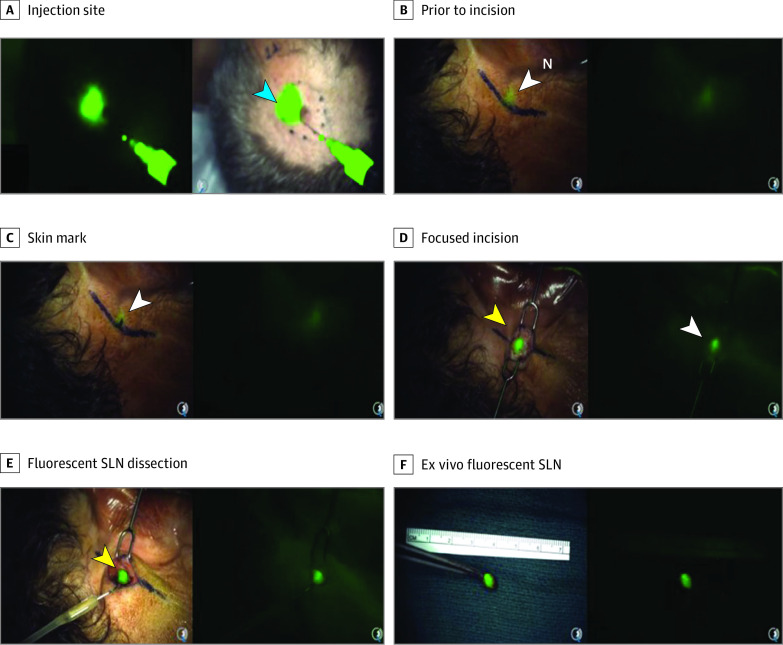
Real-Time Transcutaneous Visualization of a Postauricular Sentinel Lymph Node (SLN) Using Nanoparticles A male patient in his 50s with a scalp melanoma was injected peritumorally with integrin-targeting, dye-encapsulated nanoparticles, surface modified with polyethylene glycol chains and cyclic arginine-glycine–aspartic acid–tyrosine peptides (green signal in A). Focal fluorescence was seen through the intact skin overlying a postauricular SLN (B) using the Spectrum camera system (Quest) for real-time optical imaging guidance. Limited extent of surgical dissection (C) and size of the resection cavity (D) relative to the planned area of dissection unaided (ie, marked line in panel C is approximately 3 times larger than that actually drawn on the basis of the particle signal). Images are derived from the intraoperative video (Video).

Representative cases are shown in Figure 1 and Figure 2 as well as eFigure 2 and eFigure 3 in Supplement 2. In 2 cases (Figure 1; eFigure 2 in Supplement 2), the surgical approach used to guide nodal resection was modified earlier in the SLN biopsy procedure than would otherwise be possible due to nanoparticle–aided fluorescence detection of deep-seated (ie, approximately 1.5-2 cm deep) SLNs. In these instances, detection of SLNs in the parotid gland was achieved without injury to facial nerve branches in the vicinity of the node and without extensive nerve dissection prior to SLN localization. In Figure 2 and eFigure 3 in Supplement 2, the length of the surgical incision and extent of surgical dissection were reduced due to visualization of SLNs transcutaneously.

## Discussion

Malignant melanoma is among the fastest rising cancers in the United States, with approximately 100 350 new cases diagnosed in 2020, resulting in 6850 deaths.^21^ Head and neck melanoma is a life-threatening orphan disease with 56.1% survival at 10 years.^22^ As the presence of metastases in regional lymph nodes is a vital prognostic factor for melanoma, the NCCN recommends wide excision and SLN biopsy for patients with stage IB or II disease.^23,24^ However, using standard methods for SLN mapping, false-negative rates range from 0% to 34% (mean, 12.5%), with rates higher than those for non–head and neck melanomas.^25,26^

Current SLN practice for head and neck melanoma presents several unique challenges including (1) complex lymphatic drainage with multiple primary channels and SLN sites; (2) technical difficulties associated with nodal excision, given that short distances between primary lesion and SLNs can make detection and isolation difficult; (3) high risk of facial nerve injury, given that 25% to 30% of SLNs are found within the parotid gland area; and (4) lack of reliable, optical visualization for discriminating SLNs from adjacent fibrofatty tissue intraoperatively.^27,28^ Radioactivity guidance alone may be adequate for SLN identification in the groin or axilla where the lymphatic drainage is not as complex as in the head and neck; in the latter case, intraoperative visual guidance is extremely helpful. Importantly, gamma probes used intraoperatively to localize radiotracer accumulation often lack sufficient spatial resolution to discriminate SLNs, particularly deeper-lying nodes and/or those close to the primary tumor.^29,30,31^ Moreover, the very small size of many nodes (ie, <4 mm) makes them difficult to identify exclusively by radio-guided surgery.

Some of the limitations of radio-guided SLN localization have been addressed, in part, by traditional use of vital dyes (ie, isosulfan blue or methylene blue), but these dyes are not as sensitive as the radiotracer technique in the head and neck, with reports indicating inconsistent drainage pathways that create challenges in tracing blue channels to SLNs^32^ and that SLNs are less intensely stained than those in other body regions.^33^ Other known limitations include needing to dissect overlying soft tissue to directly visualize the blue node^34,35,36^ and risk of anaphylactoid reactions and permanent tattoos. NIR fluorescent dyes, such as indocyanine green, have also been used as an adjunct to radioguidance^37,38^ or as a hybrid fluorescent-radioactive tracer^38,39^ but have provided only incremental benefit over conventional radio-guided localization.^40,41,42^

To address these limitations, we developed a targeted Cy5.5 dye-encapsulating silica nanoparticle with an approximately 6-mm diameter (cRGDY-PEG-Cy5.5-nanoparticles), which exhibited favorable physicochemical and biological properties for image-guided surgical applications. Translation of this product to the clinic for fluorescence-guided SLN biopsy in head and neck melanoma patients was based on extensive in vivo biological and safety data,^43,44^ including a phase 1 first-in-human positron-emission tomography (PET) study.^43^ In an M21 human melanoma xenograft model, particle radiolabeling with zirconium 89 (^89^Zr)^45^ and iodine 124 (^124^I)^17^ yielded a renally clearable, dual modality (PET optical) imaging tracer showing target-or-clear detection capabilities and high TB ratios. Relatively uniform particle tracer distribution and retention were also observed at sites of disease.^45^ In addition, higher sensitivity and specificity were found for radioiodinated nanoparticles compared with the standard-of-care radiopharmaceutical ^18^F fluorodeoxyglucose (^18^F-FDG) in a spontaneous melanoma miniswine model.^5^ In miniswine, PET-avid metastatic SLNs not detected by ^18^F-FDG were identified following injection of the dual-modality product around the primary lesion.^5,44^ More recently, fluorescence-based multiplexing in the same model highlighted the excellent optical imaging capabilities of dual-modality cRGDY-PEG-Cy5.5-nanoparticles for localizing metastatic nodes with varying tumor burden as well as for assessing tumor heterogeneity.^46,47,48^ Its high brightness yielded pronounced tumor-bearing node-to–normal tissue contrast well below the microdosing level (ie, <30 nmol). Using a high-resolution (ie, <50 microns) multichannel fluorescence camera system, SLNs bearing micrometastases (ie, <2-3 mm deposits^47,48^) could also be detected, underscoring the benefit of combining such a high-resolution, high-sensitivity real-time intraoperative imaging strategy with preoperative PET imaging. Importantly, both PET and optical imaging parameters (ie, PET TB ratios; total fluorescence signal) were found to be strongly correlated with nodal tumor burden.^46^

The results of our pilot study show that the use of cRGDY-PEG-Cy5.5-nanoparticles, in conjunction with real-time intraoperative optical imaging guidance, is a safe, reliable, and clinically feasible procedure for the accurate and sensitive detection of SLNs in patients with head and neck melanoma. Our findings suggest that this particle-based technology overcomes a number of existing limitations associated with currently approved SLN contrast agents, ie, radiolabeled SC or tilmanocept, blue dye, and indocyanine green, while achieving clinically meaningful end points, including (1) favorable safety profile with high-contrast visualization using very low nanomole dosing regimens; (2) high concordance rate with technetium Tc 99m sulfur colloid, and (3) the ability to tailor surgical approach and/or reduce the extent of planned tissue dissection while (4) minimizing risk of nerve injury within the field of surgical dissection.

Based on optimization of the nanoparticle dosing strategy, we were able to obtain very high TB ratios of up to 40 at the low (nanomole) doses used. This level of contrast is not achievable with small-molecule dyes, such as indocyanine green or blue dye, which require substantially higher doses (ie, micromoles) for nodal detection. Unlike the ultrabright Cy5.5 dye-encapsulating nanoparticles, which can be optically detected as deep as approximately 2 cm below the skin, the indocyanine green signal is weakly intense at any significant depth, with limited penetration of only several millimeters, and has rapid extravasation into surrounding tissues, with marked reductions in tissue contrast.

Importantly, while we did observe lower optical signal in many benign SLNs vs the few metastatic SLNs, larger sample sizes would be needed to establish the statistical significance of this finding and to establish disease-burden thresholds for detectability. Development of tumor-specific probes that can selectively target melanoma is a highly desirable goal given the results of the second Multicenter Selective Lymphadenectomy Trial, in which no difference in melanoma-specific survival was found between the group of patients who had a positive SLN and underwent completion lymphadenectomy and those who had a positive SLN and underwent observation. Completion lymphadenectomy is no longer routinely recommended,^49^ although this same trial showed a slightly lower disease-free survival and an increased rate of regional disease control in patients who underwent completion lymphadenectomy. These findings underscore the importance of sensitively identifying and removing metastatic nodes with less invasive and more focused procedures.

It is expected that SLNs should accumulate cRGDY-PEG-Cy5.5-nanoparticles in a time-dependent manner because of mechanical inflow of the tumor-draining lymphatics, acknowledging that specific binding to cancerous cells within the node will depend on a number of factors that include binding affinity, extent of localization, and whether enough time has elapsed for washout of unbound particles from the node. While integrin-targeted nanoparticles may not be entirely tumor-specific (as is true of any nodal mapping agent), this remains an area of active investigation in our studies as we attempt to determine the association between nodal localization of nanoparticles in patients with head and neck melanoma and nodal tumor burden.

A primary end point of the study rested on the need for accurate intraoperative detection of SLNs identified on preoperative ^99m^Tc lymphoscintigraphy. High concordance (90%) was found between the proportion of ex vivo SLNs identified by technetium Tc 99m sulfur colloid and that optically visualized by cRGDY-PEG-Cy5.5-nanoparticles following early optimization procedures. Importantly, in 1 case, the SLN could not be detected in situ using technetium Tc 99m sulfur colloid because its location made it difficult to use a gamma probe, but it was able to be visualized optically using the particles. In all patients, nanoparticles led to high conspicuity of the draining tumor lymphatics and SLNs. This visual aid was considered a significant improvement by the surgeon compared with conventional radiocolloid-based gamma probe detection methods that are strictly based on count rate–related audible signals.

Visual cues provided by nanoparticles also led to alterations in surgical approach in several cases. With detection of optical signal due to depth penetration beyond 1 cm, optically bright nodes within the parotid gland could be resected based on their location without first identifying and dissecting the main trunk or divisions of the facial nerve. Thus, this nanoparticle-based image-guided surgical procedure was beneficial in terms of reducing the extent of dissection and operative time under anesthesia that otherwise would have been needed using gamma probe–aided ^99m^Tc dissection alone. The latter technique also poses significant challenges because the SLN cannot always be easily identified with a gamma probe, leading to an often lengthy surgical procedure. For the head and neck region especially, such extensive dissections can result in an increased risk of significant morbidities for these patients. In some of our other cases, the extent of the initial skin incision and subsequent tissue dissection was reduced given the more superficial location of optically identifiable SLNs that could be visualized through the intact skin prior to incision. For procedures in which the SLNs could be detected and removed, we estimated 30% to 50% reductions in the duration of the surgery using cRGDY-PEG-Cy5.5-nanoparticles as compared with technetium Tc 99m sulfur colloid alone. This range reflected variations in the type of procedure, depth of nodes from the skin surface, and the need for nerve dissection, in addition to other technical factors, such as equipment setup.

Our results set the stage for a larger randomized clinical trial that would overcome the aforementioned limitations in addition to facilitating comparisons between the conventional paradigm of preoperative lymphoscintigraphy with strategies that harness the all-in-one dual-modality imaging capabilities of these nanoparticles. Such a design could potentially aid preoperative treatment planning, intraoperative SLN visualization, and in the future, multiplexed detection of more than 1 marker while limiting extensive normal tissue resection. An important additional end point would include the correlation of the optical signal of ex vivo SLNs with nodal metastatic tumor burden to assess the ability of the technology to discriminate metastatic from nonmetastatic nodes. Importantly, this technology could also be applicable across several surgical fields in which SLN biopsy has been used, such as breast cancer, prostate cancer, gastric cancer, colorectal cancer, and non–small cell lung cancer.

### Limitations

This study has limitations. The study design could not quantitatively correlate gamma counts with optical signal as part of the nodal mapping procedure. Gamma counting is semiquantitative, at best, due to coarse spatial resolution, depth-dependent sensitivity, and scattered radiation, thereby making correlative assessments between nonvisualizable radioactive counts and optical signal measurements difficult, if not impossible. By contrast, PET activity is absolutely quantitative and can improve such correlative evaluations in planned near-term future work with a dual-modality (PET optical) nanoparticle platform. In addition, because these particles are extremely bright, many images were saturated, limiting quantitative analysis. In the future, measures will be introduced to minimize signal saturation and to otherwise standardize acquisition of optical images.

## Conclusions

This study found that ultrasmall fluorescent core-shell silica nanoparticles can be safely used intraoperatively at nanomole doses for accurate, reliable, and high-contrast visual identification of SLNs in patients with head and neck melanoma. To our knowledge, there is currently no available comparable alternative for fluorescence image–guided SLN biopsy. This novel particle-based technology could significantly change routine surgical practice and lead to safer intraoperative approaches overall, while achieving more consistent clinical results during SLN biopsy among surgeons of variable experience.

## Notes

Supplement 1. **Trial Protocol**

Supplement 2. **eAppendix.** Supplementary Methods **eFigure 1. **Dose Escalation Procedure in the Surgical Suite **eFigure 2. **Deep Tissue SLN Detection in the Intraparotid Region **eFigure 3. **Fluorescence Signal of a Metastatic SLN Through Intact Skin **eTable 1.** Summary of QC Test Results of Representative C′ Dot Batches **eTable 2. **Performance of cRGDY-PEG-Cy5.5-C′ Dots for Ex Vivo SLN Identification **eTable 3.** Comparison of cRGDY-PEG-Cy5.5-C′ Dots to Standard of Care **eReferences.**

Supplement 3. **Data Sharing Statement**
