# Supplementary material for: Use of Ultrasmall Core-Shell Fluorescent Silica Nanoparticles for Image-Guided Sentinel Lymph Node Biopsy in Head and Neck Melanoma: A Nonrandomized Clinical Trial
Source: JAMA Netw Open. 2021 Mar 18;4(3):e211936. doi: 10.1001/jamanetworkopen.2021.1936 (PMC7974643; doi:10.1001/jamanetworkopen.2021.1936)
Supplement: Supplement 1. — Trial Protocol [file jamanetwopen-e211936-s001.pdf]

Table of Contents

1.0 PROTOCOL SUMMARY AND/OR SCHEMA 3
2.0 OBJECTIVES AND SCIENTIFIC AIMS 5
3.0 BACKGROUND AND RATIONALE 6
4.0 OVERVIEW OF STUDY DESIGN/INTERVENTION 9
4.1 Design 9
4.2 Intervention 9
5.0 THERAPEUTIC/DIAGNOSTIC AGENTS 11
6.0 CRITERIA FOR SUBJECT ELIGIBILITY 11
6.1 Subject Inclusion Criteria 11
6.2 Subject Exclusion Criteria 12
7.0 RECRUITMENT PLAN 12
8.0 PRETREATMENT EVALUATION 12
9.0 TREATMENT/INTERVENTION PLAN 12
10.0 EVALUATION DURING TREATMENT/INTERVENTION 13
11.0 TOXICITIES/SIDE EFFECTS 13
12.0 CRITERIA FOR THERAPEUTIC RESPONSE/OUTCOME ASSESSMENT 13
13.0 CRITERIA FOR REMOVAL FROM STUDY 14
14.0 BIOSTATISTICS 14
15.0 RESEARCH PARTICIPANT REGISTRATION AND RANDOMIZATION PROCEDURES .. 14
15.1 Research Participant Registration 15
16.0 DATA MANAGEMENT ISSUES 15
16.1 Quality Assurance 15
16.2 Data and Safety Monitoring 16
17.0 PROTECTION OF HUMAN SUBJECTS 16
17.1 Privacy 17
17.2 Serious Adverse Event (SAE) Reporting 17
18.0 INFORMED CONSENT PROCEDURES 17
19.0 REFERENCES 18
20.0 APPENDICES 21

1.0 PROTOCOL SUMMARY AND/OR SCHEMA

This sentinel lymph node (SLN) mapping clinical trial, an extension of our previous human study (IND# 110375) demonstrating safety, will assess the utility of ultrasmall (~6 nm hydrodynamic diameter) fluorescent core-shell silica nanoparticles (C dots) for detecting disease spread to local/regional nodes in metastatic melanoma patients. The particles are non-toxic, non-aggregating, and are efficiently excreted by the kidneys, as their size is smaller than that estimated for the renal cut-off (~10 nm diameter). The core contains more than one near-infrared (NIR) fluorescence dye molecules, Cy5.5 (excitation/emission max: 678 nm/695 nm), which are covalently incorporated into an amorphous silica matrix or shell to prevent dye leaching[1, 2] and to enhance photophysical features (brightness, photostability). Measured brightness of C dot preparations is several orders of magnitude greater than that found for the parent dye in aqueous solutions; these properties render C dots ideal for optical imaging applications. The silica shell is coated with neutral poly-(ethylene glycol (PEG) chains, which has previously enabled attachment of multiple alpha-nu-beta3 ($\alpha_v\beta_3$) integrin-targeting moieties (i.e., Arg-Gly-Asp-Tyr; cRGDY) and radiolabels (^{124}I) to create a highly functionalized nanoparticle tracer[2-5], ^{124}I-cRGDY-PEG-C dots, for clinical cancer diagnostics[1]. This coat promotes bulk, but not excessively rapid, renal clearance in small animal melanoma models[6] and humans (Appendix 1b, "Urine") following intravenous (i.v.) administration, while additionally limiting recognition by the reticuloendothelial (RES) system. The renal clearance rate is optimal for targeting, as there is sufficient persistence in blood to promote tumor localization, but also rapid enough to enable prompt reductions in background activity.

A prior first-in-human PET study has been conducted at MSKCC using the ultrasmall inorganic particle tracer, ^{124}I-cRGDY-PEG-Cy5-C dots, in a cohort of 5 terminal metastatic melanoma patients. In addition to assessing particle safety, a primary objective was to quantify time-dependent whole-body tissue distributions and radiation dosimetry of ^{124}I-cRGDY-PEG-Cy5-C dots by PET imaging following intravenous (i.v.)-injection. We sought to determine whether the *in vivo* properties of this dual-modality platform (i.e., efficient clearance, low background signal, stability, bio-safety, and acceptable dosimetry) confirmed findings in our prior preclinical melanoma models. The results of these initial patient studies suggested that the particle is safe; no adverse events occurred. Pharmacokinetic (PK) profiles were found to be highly atypical for nanoparticles having sizes greater than estimated renal cut-off values (i.e., ~10 nm i.d.). Both PK and dosimetry for this particle tracer were similar to those found for other commonly used diagnostic radiotracer agents **(see Appendices 1 and 2)**, noting predominant renal clearance without appreciable particle tracer accumulations in liver, spleen, and bone marrow. In addition, dosimetric findings in humans were equivalent to that found in our preclinical melanoma models. A small fraction of the dose was seen as uptake in the stomach and salivary glands; this was presumed to be free iodine, which progressively cleared over the imaging period. The remainder of the dose cleared as an intact radioiodinated particle on the basis of radioTLC of urinary specimens. Estimated whole body half-time clearance times ranged from 13-21 hours. No significant alterations were seen in the patients' laboratory findings, including their clinical chemistries and hematologies.

We are now proposing a new first-in-human pilot study in which we utilize the optical component of non-radioactive, fluorescent cRGDY-PEG-Cy5.5-C dots, in conjunction with a hand-held, state-of-the-art fluorescence camera system, following their intradermal, peritumoral injection to determine whether this system can identify fluorescent SLN/s in a cohort of melanoma patients (n=10), as compared to standard of care radiocolloid. In support of this pilot study, we have conducted non-clinical safety studies in larger animal (miniswine) models **(see Appendix 3)**. Feasibility will be determined on the basis of achieving adequate image contrast for detection, as defined by signal-to-background ratios, and whether optical signal distinguishes diseased SLNs from non-diseased ones **(see Appendix 4)**. A longer-term objective of these pilot studies is to develop and optimize this

molecularly targeted probe as an 'all-in-one' platform for pre-operative and intraoperative mapping of local and systemic disease spread using multimodal imaging approaches.

This study will involve very limited human exposure and no diagnostic or therapeutic intent. Initiated at MSKCC under an IND application cleared by the FDA, *it will be conducted as a dose escalation procedure at least 2 hours post-injection of the radiocolloid to determine the minimum microdose needed to achieve adequate contrast for imaging nodal disease*.

In the surgical suite, subjects will undergo optical imaging after particle injection using a *hand-held* NIR fluorescence camera system (Artemis^{TM}, Middenmeer, Netherlands), a non-significant risk device, to track the real-time flow of particles within the draining tumor lymphatics and SLN/s. Intradermal particle administration about the tumor site will be administered over pre-defined dose ranges and injection volumes **(see Appendix 4)** to determine the minimum microdose needed to optimize tissue contrast in diseased nodes ; initial estimates were based on our experience in both small and large animal melanoma models. *In short, the final cRGDY-PEG-Cy5.5-C dots product will be further prepared by the investigational pharmacy as 0.5 ml injectable volumes* (in 0.9% NaCl) *to achieve a final total dose that falls within a pre-set rangeof 0.25 – 1.2 nanomoles.*. ***Technical adjustments to the particle dose will be made on a patient-by-patient basis to maximize optical signal by evaluating a range of particle concentrations and volumes (see Appendix 4).***

As particle radiolabeling will not be performed, there is no radiation limit to dosing. Preclinical studies previously performed in mice predicted the safety of non-radiolabeled, stably-iodinated (^{127}I) (^{127}I)-cRGDY-PEG-Cy5-C dots at iv doses of 800x human dose and of (^{127}I)-PEG-Cy5-C dots (without iodine moiety) by subdermal injection in a mouse tumor model at 400x the human dose on a per gram basis.

Please note that all patients entered on this protocol will undergo standard of care management in the preoperative setting using ^{99m}Tc-technetium sulfur colloid sentinel node mapping for their tumor prior to the administration of non-radioactive cRGDY-PEG-Cy5.5-C dots intraoperatively. The information obtained from the use of cRGDY-PEG-Cy5.5-C dots will not be used to guide patients' treatment planning on this protocol or any other aspects of patients' management.

Schema:

The overall objective of this study is to perform first-in-human SLN mapping studies in a small patient cohort, comparing standard of care, preoperatively administered technetium-99m (^{99m}Tc) sulfur colloid with intraoperatively injected fluorescent cRGDY-PEG-Cy5.5-C dots (0.1 – 0.5 ml over a dose range of ~0.25 to ~1.2 nanomoles in 4 quadrants about the primary lesion) for identification of sentinel nodes. Intraoperative NIR fluorescence imaging will be performed using a state-of-the-art hand-held fluorescence camera system (f 2.4) for real-time optical visualization of nodal disease, along with gamma counting for ^{99m}Tc sulfur colloid, to determine if concordance can be established as a continuous endpoint. Results will be histologically confirmed in resected tissue specimens. *Preclinical toxicology studies have demonstrated that a locally administered dose 2500 times the proposed highest human dose (1.2 nanomoles), on a nanomole per gram basis, did not induce adverse effects* (see IND section 8: Pharmacology and Toxicology and attached final report and appendix, acute toxicity testing of **non-radiolabeled** cRGDY-PEG-Cy5.5-C dots).

a. Recruitment and consent of 10 melanoma patients meeting all inclusion criteria;
b. Perform preoperative ^{99m}Tc-technetium sulfur colloid studies per routine by the nuclear medicine physicist on the day of surgery, acquiring SPECT/CT images about 0.5 – 2 hrs post-injection.
c. In the operating suite, up to 0.5 ml of cRGDY-PEG-Cy5.5-C dots, ranging in dose from about 0.25 to 1.2 nanomoles, will be injected intradermally by the surgeon about the tumor site with the skin intact, followed by gentle massage of the site (2–5 minutes) and acquisition of real-time optical images immediately after surgical exposure;

d. SLNs, both in situ and following excision, will be evaluated for evaluating NIR fluorescence and radioactive emissions from the radiocolloid, ^{99m}Tc sulfur colloid, to determine whether adequate image contrast can be achieved on the basis of measured signal-to-background (SBR) ratios. SBR will be calculated as NIR fluorescence signal of the SLN divided by signal autofluorescence of adjacent non-nodal tissue;
e. Vital signs will be monitored per routine prior to and following particle administration;
f. Biopsy or resection of SLN/s will take place per routine;
g. Histopathological and molecular characterization of excised nodes: standard of care pathologic evaluation (i.e., H&E staining and immunohistochemistry) and alpha-nu-beta3 ($\alpha_v\beta_3$) integrin expression.
h. Concordance between the gamma counts and optical signal will be assessed as a continuous endpoint using empirical (Pearson) and rank correlation (Spearman) methods.
i. Blood and urine specimens will be obtained during the first post-operative visit for standard biochemical analyses and blood counts.

2.0 OBJECTIVES AND SCIENTIFIC AIMS

The objective of this first in human SLN mapping study is to assess the feasibility of utilizing optical imaging technologies− FDA, IND-approved non-radioactive cRGDY-PEG-Cy5.5-C dots coupled with a state-of-the-art, portable fluorescent camera system- for SLN detection in the intraoperative setting. Detected optical signal will be confirmed with measured radioactive emissions using standard of care approaches. Feasibility will be investigated in the primary aim and first secondary aim.

Primary Aim: Assess whether locally injected non-radioactive cRGDY-PEG-Cy5.5-C dots injected about the primary tumor site, along with a handheld NIR fluorescence camera system, can identify fluorescent SLN/s within the nodal basin of melanoma patients intraoperatively, as determined by pathological examination.

Given the exploratory nature of this trial, the following secondary aims are proposed to address several study unknowns that relate to minimum particle dosing and the achievement of adequate optical signal in SLNs. The collection and evaluation of tissue and biological fluid specimens may permit us to better define these study unknowns.

Secondary Aim 1: Determine whether a NIR fluorescence signal-to-background ratio (SBR) of 1.1 or greater optimizes image contrast and enables identification of diseased nodes, along with the smallest dose of cRGDY-PEG-Cy5.5-C dots needed to achieve this threshold ratio.

Secondary Aim 2: Correlate optical imaging findings of in situ and excised nodal specimens, in terms of intensity, with routine gamma counting (see Section 4.2).

Secondary Aim 3: Evaluate and compare blood and urine chemistries obtained at the first post-operative visit with standardized normal values to verify that no demonstrable alterations might be attributable to particle administration.

3.0 BACKGROUND AND RATIONALE

3.1 Introduction

First-in-human pilot clinical cancer trials of molecularly targeted agents, such as cRGDY-PEG-Cy5.5-C dots, in surgical settings may serve as proof-of-concept investigations at an early stage of probe evaluation to expeditiously identify promising agents for further, large-scale intraoperative development, and to identify potential failures. *Building on our previous first-in-human PET study, this second optically-driven exploratory study will enable real-time, critical human optical data of nodal disease to be assessed earlier in the evaluation process using a different route of administration (i.e., local) relative to standard of care molecular tracer methods. Such early studies will support the design and development of more advanced phase SLN mapping trials.* Implicit in the rationale of these studies is that low particle doses (i.e., micro/nanodoses) will produce no pharmacologic or other demonstrable biologic effects by definition.

For the study proposed herein, and in consultation with Medical Physics, a decision was made to remove the C dot radiotracer component from the study design, as "downscatter" from ^{124}I high energy photons would be expected to contribute to overall radioactive counts detected in the low energy window of ^{99m}Tc. The use of these two gamma-emitting radiolabels could potentially confound interpretation of measured ^{99m}Tc sulfur colloid counts. However, as only a small fraction of cRGDY-PEG-C dots are typically radiolabeled with I-124 (private communication, Jason Lewis, Head of Cyclotron/Radiochemistry Core), the elimination of this component would not be expected to significantly alter the physicochemical and biological properties of non-radiolabeled particles.

The use of ultrasmall, fluorescent C dot preparations (<10 nm i.d.), modified for tumor targeting, and coupled with real-time NIR optical imaging, may confer the following benefits in a surgical setting: (1) more sensitive interrogation of smaller caliber lymphatic channels and/or subcentimeter nodal metastases, potentially enabling more accurate staging relative to that observed with larger-sized probes; (2) real-time optical discrimination of sentinel nodes from adjacent neurovascular structures to reduce the risk of injury during surgical procedures; and (3) enhanced clearance rates from the site of injection relative to larger-sized standard-of-care colloidal tracers[6]. The silica surface further provides a versatile template for the attachment of many small peptides and other targeting molecules that do not substantially increase C dot size, promote significant opsonization, or substantially alter renal clearance. The attachment and/or incorporation of multiple specific molecular moieties can amplify probe potency and selectivity for the target and induce multiple simultaneous interactions between the surface of the particle and the surface of the cell[7]. As such, this particle platform may offer distinct advantages for certain clinical applications over simple molecular constructs, while at the same time providing complementary biologic information at both the tissue and cellular levels.

3.2 Sentinel lymph node mapping and biopsy: current clinical practice

SLN mapping techniques, routinely used in staging melanoma, specifically identify the node/s that drain the primary tumor and are at highest risk of tumor metastases. The identification of lymph node metastases permits patients to be stratified to appropriate treatment arms in a more timely fashion, thereby potentially improving patient outcomes.

Standard-of-care SLN mapping techniques rely on the uptake of an imaging agent, injected about the primary tumor site, for transport to the SLN via one or more lymphatic channels. One such agent, filtered ^{99m}Tc-sulfur colloid, is injected pre-operatively around the primary tumor for SLN localization and visualized with a gamma camera co-registered to a CT scan for spatial orientation. Intraoperatively, a hand-held gamma probe is used to measure radioactivity in the draining lymphatic structures, and help the surgeon localize the sentinel lymph node. SLN mapping with ^{99m}Tc-sulfur colloid radiotracer is standard-of-care procedure for staging the regional nodal basin in early

melanoma (~50,000 procedures per year in US). Another intraoperative adjunct for localizing SLNs is isosulfan (Lymphazurin 1%, US Surgical, North Haven, CT) or ‘blue dye’, which turns the SLN blue following injection into the peritumoral region and allows visual identification of a “hot and blue” SLN.

Current SLN mapping and biopsy techniques suffer from several drawbacks. Primarily, spatial resolution is low, offering no real time visualization or detailed anatomy of nodes and lymphatic channels within the operative field. In addition, filtered ^{99m}Tc-sulfur colloid particles, ranging in size from 10-100 nm, demonstrate slow clearance from the site of injection (i.e., interstitial space), which can effectively limit adequate visualization of the draining lymphatics. Although the SLN may be radioactive and “hot” to the intraoperative gamma probe, the operating surgeon needs to rely principally on an abnormal visual appearance and palpation to discriminate the SLN and reliably differentiate it from adjoining tissues. Moreover, intraoperative identification of SLNs which can be 4-5mm in size is fraught with the risk of injury to important adjacent structures such as nerves and vessels. Within certain areas of the body such as the head and neck, injury to neurovascular structures can result in dramatic consequences and can permanently alter functions such as speech and swallowing, and the cosmetic appearance of the patient. Within the head and neck, failure to identify any drainage pattern or localize small nodes occurs in up to ~10% of cases. Staging of head and neck melanomas has been hampered by unpredictable patterns of metastatic disease spread, difficult-to-detect nodes in anatomic proximity to tumor, and difficulty in intraoperative differentiation of small nodes from vital structures during surgery. <u>If adjunctive blue dye injection is used, the blue SLN is apparent only if it is located superficially in the operative field and may not become apparent until significant amount of tissue dissection has taken place.</u> *The foregoing limitations associated with standard-of-care SLN mapping techniques, along with innovations in agent development and imaging technologies, have spurred efforts to develop new tools for improving lymphatic imaging strategies and identify the SLN for biopsy*. ***In this first-in-human SLN mapping trial, blue dye will be replaced with fluorescent cRGDY-PEG-Cy5.5-C dots incorporating the NIR dye, Cy5.5.***

Although traditional NIR organic dyes (e.g., Cy7, Cy5.5) are frequently used to map the lymphatic system, these probes have associated drawbacks. Dyes are prone to extravasation into the surrounding tissues given their small size and require conjugation to macromolecules (i.e., proteins, immunoglobulins) for retention within the lymphatic system. Their reduced brightness and photostability decrease useful imaging penetration depths, and their relatively wide emission spectra can result in destructive spectral interference, precluding their use in multispectral imaging applications[7]. The FDA-approved NIR dye indocyanine green (ICG, emission peak ~830 nm) is a commonly used fluorophore in clinical settings[8, 9] to image lymphatic flow and SLN/s at very low doses[9]. However, the versatility of this agent is limited, and the absence of functional groups can make conjugation to targeting and/or contrast-producing moieties challenging[10]. Given the weak and unstable nature of this NIR dye, depth penetration is restricted, with detection largely confined to interrogation of superficial nodes.

For lymphatic imaging, ideal imaging agents should exhibit key properties that improve SLN tissue localization and retention (i.e., surface receptor binding, internalization), enhance imaging signal at the target site, and promote more rapid clearance from the site of injection and the body in order to maximize target-to-background ratios (i.e., agent should target and clear)[11]. For mapping lymphatic tumor spread using a particle-based agent, key design constraints need to be met to achieve maximum diagnostic/therapeutic benefit while minimizing associated complications (i.e., injury to adjacent critical structures, lymphedema).

3.3 Targeted Multimodality C dots

For in vivo cancer diagnostic applications, including tumor targeting, monitoring of metastatic tumor spread, and assessment of metastatic (vs reactive) lymph nodes, target-specific molecular or nanoparticle probes are needed. The integrin $\alpha_v\beta_3$ plays an important role in angiogenesis, survival, and metastatic tumor spread[12]. It is expressed on activated endothelial cells, as well as some tumor cells, including melanoma. Thus, it is considered a promising imaging target, acting as a potential surrogate for assessing neoangiogenesis. Molecular imaging of $\alpha_v\beta_3$ expression could potentially facilitate response evaluation of antiangiogenic drugs (e.g., bevacizumab) or aid in selecting and monitoring patients receiving humanized monoclonal antibody therapies directed against $\alpha_v\beta_3$ (EMD121974)[12]. While many different imaging technologies have been utilized for performing preclinical studies evaluating $\alpha_v\beta_3$ expression, PET and SPECT, using the integrin receptor-specific <u>peptide</u> tracers [^{18}F]galacto-RGD[12] and [^{99m}Tc]NC100692, are the only techniques which, up to now, have been successfully used to diagnose disease in patients[13].

We have previously performed *in vivo* SLN mapping studies using PET and optical imaging approaches following attachment of a cyclic tripeptide sequence, Arg-Gly-Asp-Tyr (cRGDY)[14-20], to either radiolabeled[11] or non-radiolabeled[6] Cy-5 containing cRGDY-PEG-C dots, for detection of $\alpha_v\beta_3$ integrin expression on activated endothelial cell and/or tumor cell surfaces in melanoma models during angiogenesis[5]. The majority of preclinical studies to date have performed integrin expression imaging of $\alpha_v\beta_3$ using RGD peptide- or peptide-conjugate tracers as targeting ligands[16, 21-23], which in many cases, bind to $\alpha_v\beta_3$ integrins expressed on tumor cells and/or tumor neovasculature[24].

Newer-generation molecular and particle-based agents, such as non-targeted activatable[8, 10, 25] and targeted organic fluorophores[26-28], gadolinium labeled dendrimers[29-31] and other nanocarriers[32], and macromolecular agents[33-35], have been developed for use with image-guided procedures, such as SLN mapping, as detailed in a number of comprehensive reviews[10, 31, 36-42]. The more recent introduction of multimodal nanoparticles[6, 43, 44] for use with at least two imaging modalities can potentially improve lymph node resection efforts by aiding pre-operative planning and intraoperative guidance on the basis of a single platform technology. Such dual-modality agents, coupled with increasingly sensitive and higher resolution portable optical imaging devices enable improvements to overall imaging quality to be achieved in real-time by making adjustments to signal gain, exposure time, and/or background signal levels during image acquisition, as well as by performing signal stretching. Such adjustments permit image-guided treatment to be placed under the direct control of the operating surgeon. Typically, such applications have utilized particle-based agents which are modestly larger (i.e., 40 - 100 nm diameter) than antibodies and significantly larger than low-molecular-weight pharmaceuticals [7] to image integrin expression involving tumor neovasculature[45-51].

By contrast, the targeted C dot proposed for this pilot study measures, on average, 6 nm i.d, which is roughly comparable to the average diameter of a protein molecule, albumin, and smaller than the estimated renal cut-off value. Relative to free dyes, the larger overall size of these macromolecular-size particle probes render them less prone to extravasation, lead to extended circulation half times (~hours), and are associated with increased probe bioavailability, facilitating tumor targeting and retention.

3.4 Rationale

Given the favorable physicochemical, biological, and dosimetric properties of these ultrasmall, neutrally charged, integrin-targeted C dots in both preclinical models and humans, along with prior toxicology assessments, this targeted probe was selected for testing in a small cohort (n=10) of melanoma patients. This macromolecular-size probe offers several advantages over conventional imaging probes, including (1) enhanced receptor binding affinity/avidity that arises from multiple simultaneous interactions between the particle and cell surface (e.g., $\alpha_v\beta_3$ integrin) receptors, and (2) improved SLN tissue localization and retention, target-to-background ratios, and clearance from the

site of injection and the body. Although targeted C dots are larger in size than low- molecular weight radiopharmaceuticals, they are considered of intermediate or macromolecular-size relative to antibodies and typical particle probe standards (10 – 100 nm i.d.). The macromolecular-size of the radiolabeled targeted C dot improves upon the characteristically slow tissue diffusion and/or transport through delivery barriers associated with larger-sized particle probes.

4.0 OVERVIEW OF STUDY DESIGN/INTERVENTION

4.1 Design

This is a one-year pilot SLN mapping feasibility study that will enroll 10 newly diagnosed or recurrent melanoma patients at MSKCC. Pre-operative standard-of-care SPECT/CT imaging findings of the primary melanoma and draining lymph node basin will be used to optimize patient positioning in the operative suite prior to conducting fluorescence imaging and surgical excision of the primary lesion and SLN/s. An intraoperative, hand-held multichannel fluorescence camera system will acquire two-dimensional large field-of-view NIR images in the context of adjacent soft tissue structures for assessing the full extent of nodal disease within the nodal basin. The camera system will be optimized for real-time, detection of Cy5.5 optical signal during SLN mapping procedures in humans. Confirmation of fluorescence signal from tumor and nodes within the surgically exposed nodal basin will be based on measurements of low-energy gamma emissions from ^{99m}Tc-sulfur colloid using routine gamma probes. These probes will be used to determine counts at the tumor site, identify SLN/s and suspected metastatic nodes, as well as assay background tissues.

Non-radioactive cRGDY-PEG-Cy5.5-C dotswill be injected intradermally in 4-quadrants about the tumor site into intact skin, following by high resolution optical scanning in video mode (>15 frames per second) to map dynamic changes in particle transit from the injection site into the draining lymphatics and nodes with successive surgical exposure. *The maximum total dose injected will be 1.2 nanomoles.. This* cRGDY-PEG-Cy5.5-C dot *dose was also locally administered about murine melanoma xenografts as part of our single dose subcutaneous injection toxicity study-equivalent to approximately 2500x the highest anticipated starting dose in human (1.2 nmol) on a nmol/gm basis. For this study, and as part of an in vivo dose escalation procedure, both the dose (nanomoles) and volume of cRGDY-PEG-Cy5.5-C dots may be systematically varied, up to about a factor of 5, using either a single dose or multiple dosing strategy to determine minimum microdosing requirements for enhancing optical detection of nodal disease and determining threshold signal-to-background ratios for achieving optimal tissue contrast.* Corresponding radioactivity measurements will be made in situ (and ex vivo) using ^{99m}Tc sulfur colloid; counts will be compared with the corresponding measured NIR fluorescence signal and SBRs at these sites prior to and after excising the nodes in question for pathologic evaluation. *For details regarding the design and execution of the particle dose escalation procedure, please see Appendix 4. Technical parameters (particle concentration, total injection volume, and final injected particle dose) will be adjusted and recorded on a patient-by-patient basis.*

Excised tissue specimens will be collected, counted, and assessed optically using the same procedure as above. Specimens will be histologically processed and evaluated as per standard protocol in the Department of Pathology, including routine H&E staining and immunohistochemistry for melanoma (S-100, HMB-45) and additional expression markers for $\alpha_v\beta_3$ integrin (non-routine). Histologic results will be correlated with imaging findings.

4.2 Intervention

- Ten (10) patients with newly diagnosed or recurrent melanoma will undergo a dose escalation procedure by systematically varying the dose and/or volume of cRGDY-PEG-Cy5.5-C dots locally injected about the primary tumor site to establish the minimum microdose needed for

mapping metastatic SLN/s with the hand-held fluorescent camera system with histologic confirmation. Real-time technical adjustments will be made on a patient-by-patient basis to determine threshold signal-to-background ratios needed for achieving adequate image contrast.

- A standard-of-care SLN mapping procedure will be performed in head and neck melanoma patients. All subjects will be injected peritumorally with approximately 7.4 MBq ^{99m}Tcsulfur colloid on the day of surgery, divided into 4 injections around the lesion, and gamma camera imaging will be acquired about 0.5 – 2 hrs later. Patients will then be transported to the operating suite and, prior to the procedure, will receive one or more intradermal/peritumoral injections of cRGDY-PEG-Cy5.5-C dots (up to 1.2 nanomoles total) at four sites about the lesion, as part of a dose escalation procedure (see below and Appendix 4). Injection of cRGDY-PEG-Cy5.5-C dots will be performed by the surgeon. Subsequently, gentle pumping pressure will be applied to the injection site for 1 min.

- The dose escalation procedure, described in detail in Appendix 4, can be briefly described here as follows: Both optical signal (image contrast) and flow to the SLN will be evaluated starting with the lowest particle concentration (2.0 nmoles/ml) and injectable volume (0.1 ml), as detailed below. If adequate signal is not observed upon injection about the primary lesion, the particle concentration will initially be increased, up to a maximum of 6.0 nanomoles/ml, until this condition is met. Upon establishing adequate optical signal (contrast), we will evaluate whether adequate flow to the SLN has occurred. If not, additional incremental volumes (0.1 ml increments) will be administered until the SLN is visualized. For any of these combinations, the maximum injectable dose is 1.2 nanomoles.

- Following sterile covering of the surgical field, NIR fluorescence imaging will be performed after surgical exposure of the injection site and adjacent nodal basin with the imaging head of the portable Artemis fluorescence camera system positioned at about 10-30 cm distance to the surgical field. The lights in the operating room will be turned off, and the surgical field will be illuminated (irradiance < 10 mW/cm^2; limit for skin 200 mW/cm^2) to assess fluorescence signal in the SLN. Camera exposure times will be less than 80 ms. The standard surgical procedure, including sentinel node biopsy, will otherwise be performed with the operating room lights on.

- Using the Artemis™ portable fluorescence camera system and Capture Suite, image streams will be recorded from the start of injection through to biopsy and *ex vivo* imaging in order to document lymphatic flow and accumulation of cRGDY-PEG-Cy5.5-C dots in SLNs. The NIR channel image data will be quantified and recorded by averaging three maximum pixel intensities (0-256 scale) along the length of a given SLN once sufficient fluorescence is detected in situ and then ex vivo following biopsy; device settings will also be recorded (i.e., gain, exposure time) on a case-by-case basis. Likewise, non-nodal background readings will be made approximately 1-2 cm from the SLN (pre- and post-biopsy) and recorded. A SLN exhibiting a signal-to-background ratio (SBR) of ≥ 1.1 in situ and ex vivo will be considered positive by NIR fluorescence. In the NIR region of the spectrum, background levels are essentially stable and low enough to meet this condition. Three five-second gamma probe counts will also be taken while placing the probe directly against the SLN, both in situ and ex vivo.

- Excised tissue specimens will be collected and processed as per standard protocol in the Department of Pathology, including routine H&E staining and immunohistochemistry for melanoma (S-100, HMB-45), as well as $\alpha_v\beta_3$ integrin expression markers (non-routine).

- Blood and urine specimens will be collected during the first post-operative visit for evaluating and comparing chemistries (comprehensive metabolic panel (CMP), complete blood counts (CBC). and urinalysis) with normal value ranges to verify that no demonstrable changes can be attributable to particle administration.

5.0 THERAPEUTIC/DIAGNOSTIC AGENTS

The agent, cRGDY-PEG-Cy5.5-C dots, is a PEG-coated cRGD-bound macromolecular-size silica nanoparticle tracer that covalently integrates bound Cy5.5 dyes in a sol-gel derived silica matrix. The particles are assembled in a core-shell architecture via a modified Stober synthesis[52-55] with the dye molecules sequestered within the particle core, which is enclosed in a layer of pure silica. cRGDY-PEG-Cy5.5-C dots in water for injection, USP are supplied by Cornell University in Ithaca, New York and the final product is vialed and tested at MSKCC. The product is manufactured under controlled, GMP guidelines appropriate for Phase I drugs and will be accompanied by a COA as described in detail in the chemistry, manufacturing and controls section of this IND.

The investigational Pharmacy at MSKCC will further dilute the final product in in isotonic (0.9%) saline solution and dispense cRGDY-PEG-Cy5.5-C dots for dose escalation studies 2 – 3 hours before the procedure. Each concentration will be contained within an appropriately labeled 0.5-ml hospital standard sterile syringe. All syringes will be kept in a secure 2-8 centigrade refrigerator that is equipped with a chart recorder, alarm, or monitoring device until dispensed to the operating room pharmacy. We will maintain a current running inventory of drug supplies.

6.0 CRITERIA FOR SUBJECT ELIGIBILITY

6.1 Subject Inclusion Criteria

- 18 years of age or older
- Histologically confirmed diagnosis of melanoma at MSKCC
- Newly-diagnosed or recurrent (local, regional, metastatic) malignant melanoma patients in whom SLN mapping is indicated
  - Residual clinically or radiographically evident tumor, including primary cutaneous and mucosal melanomas
  - Prior radiation therapy, chemotherapy, or surgery in patients requiring flap reconstruction in the head and neck region.
  - Newly diagnosed patients with previous excisional biopsy.
- Normal baseline cardiac function based upon EKG and pre-operative evaluation
- ANC>1000/mcl and platelets>100,000/mcl.
- Bilirubin level of < 2.0 mg/dl in the absence of a history of Gilbert's disease (or pattern consistent with Gilbert’s).
- If patients have a history of malignancy other than melanoma, they must be disease-free for ≥ 5 years at the time of enrollment.
- All patients of childbearing and child-creating age must be using an acceptable form of birth control
- Women who are pre-menopausal must have a negative serum pregnancy test

6.2 Subject Exclusion Criteria

- Known pregnancy or breast-feeding.
- Medical illness unrelated to the tumor which in the opinion of the attending physician and principal investigator will preclude administration of the agent. This includes patients with uncontrolled infection, chronic renal insufficiency, myocardial infarction within the past 6 months, unstable angina, cardiac arrhythmias other than chronic atrial fibrillation and chronic active or persistent hepatitis, or New York Heart Association Classification III or IV heart disease.
- History of any malignancy other than melanoma for which the disease-free interval is <5 years and/or on active therapy for that malignancy.

7.0 RECRUITMENT PLAN

Patients will be evaluated by the attending physician from the Department of Head and Neck Surgery and the Melanoma Service, and entered onto the study if they are appropriate candidates. Potential research participants will be selected according to the above inclusion/exclusion criteria. Imaging and surgical criteria will not be used for selecting participants.

8.0 PRETREATMENT EVALUATION

All patients will receive the necessary baseline scans and tests prior to injection of C dots according to the standard of care for their melanoma as follows:

Within 2 weeks prior to treatment initiation:

- Complete history and physical
- CBC with differential
- Electrolytes (Na, K, Cl, CO2), BUN, Cr, bilirubin, total protein, albumin, alkaline phosphatase, uric acid, phosphorus, AST, ALT, LDH, urinalysis as needed
- pregnancy test in pre-menopausal women
- EKG as needed
- CT scan of the neck as needed;
- CTs of the chest, abdomen, and pelvis as needed
- PT, APTT, type &screen

9.0 TREATMENT/INTERVENTION PLAN

Agent Injection:

Pre-operatively, the patient will initially undergo routine imaging 0.5 – 2 hrs after local injection of ^{99m}Tc-technetium sulfur colloid per the standard procedure. In the operative suite, intradermal, 4-quadrant injections of cRGDY-PEG-Cy5.5-C dots will be administered as a single or multiple doses about the tumor site.

Optical Scanning Protocol:

The patient's optical scans will be acquired in the surgical suite using the Artemis™ portable fluorescence camera system (f 2.4; 15 frames per second; resolution 50 μm) and video monitoring. In the operating room, vital signs will be monitored per routine prior to and after agent administration.

10.0 EVALUATION DURING TREATMENT/INTERVENTION

Image Analysis:

Using Artemis™ Capture Suite, image streams will be recorded from the start of injection through to nodal excision and *ex vivo* imaging in order to document lymphatic flow and accumulation of cRGDY-PEG-Cy5.5-C dots in SLNs. The NIR channel image data will be quantified, either during or after the procedure, by averaging three maximum pixel intensities (0-256 scale) along the length of a given SLN once sufficient fluorescence is detected in situ and ex vivo post-resection. Likewise, non-nodal background signal readings will be made approximately 1-2 cm from the SLN (pre- and post-excision). Gamma probe counts will be performed as per standard practice.

Image post-processing will be performed in real time to correct for the contribution of background autofluorescence to the overall fluorescence signal measured in order to enhance tumor-to-background ratios and tissue contrast. The results will be kept as numeric as produced by post-processing. In addition, an investigator-assessed presence or absence of tumor in the lymph node will also be recorded.

Tissue sampling for surgical patients:

To confirm imaging findings, tumor tissue will be obtained from the biopsy or surgical sample in the operating room, after adequate specimens have been obtained for diagnostic purposes, as determined by the attending surgeon and pathologist. SLN specimens will be processed as per standard protocol in the Department of Pathology. Markers for $\alpha_v\beta_3$ integrin expression will be applied to tissue sections by the Laboratory of Comparative Pathology. Serial sections will be reviewed by an experienced pathologist.

11.0 TOXICITIES/SIDE EFFECTS

No side effects are expected as a result of this study. However, in the unlikely event that an adverse reaction to this non-radioactive particle probe occurs, the results will be documented and reported by the Principal Investigator to the Institutional Review Board. CTC version 4.0 will be used to grade toxicity.

12.0 CRITERIA FOR THERAPEUTIC RESPONSE/OUTCOME ASSESSMENT

This pilot study will evaluate the feasibility and characteristics of non-radioactive cRGDY-PEG-Cy5.5-C dots coupled with a state-of-the-art, portable fluorescent camera system- for SLN detection in the intraoperative setting. Given the exploratory nature of this trial and the early stage of fluorescent particle probe development for such indications, there are no well-established optical imaging protocols, outcome measures, or correlative data which have been established and which can be used for benchmarking against other agents. These outcome measures need to be empirically tested and validated for each probe-device combination and area of the body interrogated. Accordingly, we will assess whether locally injected non-radioactive cRGDY-PEG-Cy5.5-C dots injected about the primary tumor site, along with a handheld NIR fluorescence camera system, can identify fluorescent SLN/s within the nodal basin of melanoma patients intraoperatively, as determined by pathological examination and confirmed with measured radioactive emissions using standard of care approaches._We will measure *fluorescence signal within the node on the basis of averaging several maximum pixel intensities*; this is an optical read-out provided by the camera system, and which can, in turn, be correlated with gamma counts. Correlations will be performed for nodes in situ and ex vivo.

Previous studies with clinically-approved NIR dye indocyanine green (ICG) have found that a 1.1 signal to background ratio (SBR) enables identification of diseased nodes in breast cancer patients (*J. Sven D. Mieog et al, "Toward Optimization of Imaging System and Lymphatic Tracer for Near-Infrared Fluorescent Sentinel Lymph Node Mapping in Breast Cancer" Ann Surg Oncol (2011) 18:2483–2491*). We will determine whether this ratio is adequate over the range of particle doses used within the context of this exploratory study. Since we are also working in the near infrared part of the light spectrum with cRGDY-PEG-Cy5.5-C dots (i.e., $\lambda_{excitation}/\lambda_{emission}$ ~690/720 nm), background signal is expected to be negligible, and thus signal changes which are about 10% higher than background levels should be sensitively detected.

The Artemis™ Capture Suite will be used as an optical read-out.,Image streams will be recorded from the start of injection through to nodal excision and *ex vivo* imaging in order to document lymphatic flow and accumulation of cRGDY-PEG-Cy5.5-C dots in SLNs. The NIR channel image data will be quantified, either during or after the procedure, by averaging three maximum pixel intensities (0-256 scale) along the length of a given SLN once sufficient fluorescence is detected in situ and ex vivo post-resection. Likewise, non-nodal background signal readings will be made approximately 1-2 cm from the SLN (pre- and post-excision). Three, 5-sec gamma probe counts will also be taken while placing the probe directly against the SLN, both in situ and ex vivo.

Image post-processing will be performed in real time to correct for the contribution of background autofluorescence to the overall fluorescence signal measured in order to enhance tumor-to-background ratios and tissue contrast. The results will be kept as numeric as produced by post-processing. In addition, an investigator-assessed presence or absence of tumor in the lymph node will also be recorded.

To confirm imaging findings, tumor tissue will be obtained from the biopsy or surgical sample in the operating room, after adequate specimens have been obtained for diagnostic purposes, as determined by the attending head and neck surgeon and pathologist. SLN specimens will be processed as per standard protocol in the Department of Pathology. Markers for $\alpha_v\beta_3$ integrin expression will be applied to tissue sections by the Laboratory of Comparative Pathology. Serial sections will be reviewed by an experienced neuropathologist.

We will also verify whether there are any blood and urine chemistries alterations attributable to particle administration at the first post-operative visit compared to standardized normal values.

13.0 CRITERIA FOR REMOVAL FROM STUDY

- Although no side effects are expected as a result of this study, if severe, unexpected toxicities/side effects are experienced by any 2 patients enrolled, then the clinical trial will be stopped.
- If at any time, the patient is found to be ineligible for the protocol as designated in the section on Criteria for Patient/Subject Eligibility (i.e., a change in diagnosis), the patient will be removed from the study.

14.0 BIOSTATISTICS

We will enroll 10 patients to assess the feasibility of conducting pre-operative SLN mapping in patients with metastatic melanoma using real-time optical detection procedures and intradermal single- or double-dose injection/s of non-radioactive cRGDY-PEG-Cy5.5-C dots about the primary tumor site. Feasibility will be determined on the basis of achieving adequate image contrast for detection, as defined by signal-to-background ratios, and whether optical signal

distinguishes diseased SLNs from non-diseased ones **(see Appendix 4)**. Our sample size is chosen based on ethical, logistical and financial limitations rather than statistical considerations and is consistent with other pilot studies in the literature. Expected length of accrual is about 1 year.

All aims will be addressed with descriptive analysis. For the primary aim optical signal will be summarized as a continuous variable separately within the diseased and non-diseased lymph nodes. No statistical analysis is planned for secondary aim 1. Secondary aim 2 of correlating optical imaging findings of SLN/s, either within the nodal basin or as excised nodal specimens, with routine gamma counting will be addressed by empirical (Pearson) and rank correlation (Spearman) methods with both variables as continuous. For secondary aim 3, proportion of blood and urine lab values that fall outside the standardized ranges will be reported.

15.0 RESEARCH PARTICIPANT REGISTRATION AND RANDOMIZATION PROCEDURES

15.1 Research Participant Registration

Confirm eligibility as defined in the section entitled Criteria for Patient/Subject Eligibility.

Obtain informed consent, by following procedures defined in section entitled Informed Consent Procedures.

During the registration process registering individuals will be required to complete a protocol specific Eligibility Checklist.

All participants must be registered through the Protocol Participant Registration (PPR) Office at Memorial Sloan-Kettering Cancer Center. PPR is available Monday through Friday from 8:30am – 5:30pm at 646-735-8000. Registrations must be submitted via the PPR Electronic Registration System (http://ppr/). The completed signature page of the written consent/RA or verbal script/RA, a completed Eligibility Checklist and other relevant documents must be uploaded via the PPR Electronic Registration System.

16.0 DATA MANAGEMENT ISSUES

A Radiology Research Study Assistant (RSA) will be assigned to the study. The responsibilities of the RSA include project compliance, data collection, abstraction and entry, data reporting, regulatory monitoring, problem resolution and prioritization, and coordinate the activities of the protocol study team.

The data collected for this study will be entered into a secure database. Source documentation will be available to support the computerized patient record. All research material from this study will be handled with the same confidentiality as the patient's other medical data.

16.1 Quality Assurance

Weekly registration reports will be generated to monitor patient accruals and completeness of registration data. Routine data quality reports will be generated to assess missing data and inconsistencies. Accrual rates and extent and accuracy of evaluations and follow-up will be monitored periodically throughout the study period and potential problems will be brought to the attention of the study team for discussion and action.

Random-sample data quality and protocol compliance audits will be conducted by the study team, at a minimum of two times per year, more frequently if indicated.

16.2 Data and Safety Monitoring

The Data and Safety Monitoring (DSM) Plans at Memorial Sloan-Kettering Cancer Center were approved by the National Cancer Institute in September 2001. The plans address the new policies set forth by the NCI in the document entitled "Policy of the National Cancer Institute for Data and Safety Monitoring of Clinical Trials" which can be found at: *http://cancertrials.nci.nih.gov/researchers/dsm/index.html*. The DSM Plans at MSKCC were established and are monitored by the Office of Clinical Research. The MSKCC Data and Safety Monitoring Plans can be found on the Memorial Sloan-Kettering Cancer Center Intranet at: *http://mskweb2.mskcc.org/irb/index.htm*.

There are several different mechanisms by which clinical trials are monitored for data, safety and quality. There are institutional processes in place for quality assurance (e.g., protocol monitoring, compliance and data verification audits, therapeutic response, and staff education on clinical research QA) and departmental procedures for quality control, plus there are two institutional committees that are responsible for monitoring the activities of our clinical trials programs. The committees: *Data and Safety Monitoring Committee (DSMC)* for Phase I and II clinical trials, and the *Data and Safety Monitoring Board (DSMB)* for Phase III clinical trials, report to the Center's Research Council and Institutional Review Board.

17.0 PROTECTION OF HUMAN SUBJECTS

There are no foreseen additional risks to the patients from this study. Alternatives to participating in the study include getting treatment or care for your cancer without being in a study or taking part in another study.

Risks of Study Participation: Patients in this study will be receiving current standard of care for their specific disease site.

Financial Costs to Patients: Because all of the diagnostic and therapeutic interventions, except for the cRGDY-probe optical scans, are part of the current routine care of patients/subjects eligible for this study, and given that no costs will be incurred for optical scanning, no additional financial costs or burdens will need to be assumed by the patient beyond the charges routinely incurred as part of standard medical care.

Patient Confidentiality: Patient/subject privacy and confidentiality will be maintained according to MSKCC guidelines and all data derived from this study will be kept in a secure database. All data and results will be anonymously reported with regard to individual subjects.

Voluntary nature of the study: Subjects will be made aware of the voluntary nature of the study as part of the informed consent process. Subject participation terminates following acquisition of optical images and collection of biological specimens. However, they will be allowed to withdraw participation at any time without the risk of alteration in the quality of their medical care.

17.1 Privacy

MSKCC's Privacy Office may allow the use and disclosure of protected health information pursuant to a completed and signed Research Authorization form. The use and disclosure of protected health information will be limited to the individuals described in the Research Authorization form. A Research Authorization form must be completed by the Principal Investigator and approved by the IRB and Privacy Board (IRB/PB).

17.2 Serious Adverse Event (SAE) Reporting

Any SAE must be reported to the IRB/PB as soon as possible but no later than 5 calendar days. The IRB/PB requires a Clinical Research Database (CRDB) SAE report be submitted electronically to the SAE Office at sae@mskcc.org containing the following information:

Fields populated from CRDB:
- Subject's name (generate the report with only initials if it will be sent outside of MSKCC)
- Medical record number
- Disease/histology (if applicable)
- Protocol number and title

Data needing to be entered:
- The date the adverse event occurred
- The adverse event
- Relationship of the adverse event to the treatment (drug, device, or intervention)
- If the AE was expected
- The severity of the AE
- The intervention
- Detailed text that includes the following
  - A explanation of how the AE was handled
  - A description of the subject's condition
  - Indication if the subject remains on the study
  - If an amendment will need to be made to the protocol and/or consent form.

The PI's signature and the date it was signed are required on the completed report.

For IND/IDE protocols:
The CRDB AE report should be completed as above and the FDA assigned IND/IDE number written at the top of the report. If appropriate, the report will be forwarded to the FDA by the SAE staff through the IND Office.

18.0 INFORMED CONSENT PROCEDURES

Before protocol-specified procedures are carried out, consenting professionals will explain full details of the protocol and study procedures as well as the risks involved to participants prior to their inclusion in the study. Participants will also be informed that they are free to withdraw from the study at any time. All participants must sign an IRB/PB-approved consent form indicating their consent to participate. This consent form meets the requirements of the Code of Federal

Regulations and the Institutional Review Board/Privacy Board of this Center. The consent form will include the following:

1. The nature and objectives, potential risks and benefits of the intended study.
2. The length of study and the likely follow-up required.
3. Alternatives to the proposed study. (This will include available standard and investigational therapies. In addition, patients will be offered an option of supportive care for therapeutic studies.)
4. The name of the investigator(s) responsible for the protocol.
5. The right of the participant to accept or refuse study interventions/interactions and to withdraw from participation at any time.

Before any protocol-specific procedures can be carried out, the consenting professional will fully explain the aspects of patient privacy concerning research specific information. In addition to signing the IRB Informed Consent, all patients must agree to the Research Authorization component of the informed consent form.

Each participant and consenting professional will sign the consent form. The participant must receive a copy of the signed informed consent form.

19.0 REFERENCES

1. Burns AA, Vider J, Ow H, et al. Fluorescent silica nanoparticles with efficient urinary excretion for nanomedicine. *Nano Lett.* Jan 2009;9(1):442-448.

2. Burns A, Ow H, Wiesner U. Fluorescent core-shell silica nanoparticles: towards "Lab on a Particle" architectures for nanobiotechnology. *Chem Soc Rev.* Nov 2006;35(11):1028-1042.

3. Ow H, Larson DR, Srivastava M, Baird BA, Webb WW, Wiesner U. Bright and stable core-shell fluorescent silica nanoparticles. *Nano Lett.* Jan 2005;5(1):113-117.

4. Burns A, Sengupta P, Zedayko T, Baird B, Wiesner U. Core/Shell fluorescent silica nanoparticles for chemical sensing: towards single-particle laboratories. *Small.* Jun 2006;2(6):723-726.

5. Choi J, Burns AA, Williams RM, et al. Core-shell silica nanoparticles as fluorescent labels for nanomedicine. *J Biomed Opt.* Nov-Dec 2007;12(6):064007.

6. Benezra M, Penate-Medina O, Zanzonico PB, et al. Multimodal silica nanoparticles are effective cancer-targeted probes in a model of human melanoma. *J Clin Invest.* Jul 2011;121(7):2768-2780.

7. Montet X, Montet-Abou K, Reynolds F, Weissleder R, Josephson L. Nanoparticle imaging of integrins on tumor cells. *Neoplasia.* Mar 2006;8(3):214-222.

8. Crane LM, Themelis G, Arts HJ, et al. Intraoperative near-infrared fluorescence imaging for sentinel lymph node detection in vulvar cancer: first clinical results. *Gynecol Oncol.* Feb 2011;120(2):291-295.

9. Sevick-Muraca EM, Sharma R, Rasmussen JC, et al. Imaging of lymph flow in breast cancer patients after microdose administration of a near-infrared fluorophore: feasibility study. *Radiology.* Mar 2008;246(3):734-741.

10. Rasmussen JC, Tan IC, Marshall MV, Fife CE, Sevick-Muraca EM. Lymphatic imaging in humans with near-infrared fluorescence. *Curr Opin Biotechnol.* Feb 2009;20(1):74-82.

11. Bradbury MS, Phillips E, Montero PH, et al. Clinically-translated silica nanoparticles as dual-modality cancer-targeted probes for image-guided surgery and interventions. *Integr Biol (Camb).* Jan 2013;5(1):74-86.

12. Hood JD, Cheresh DA. Role of integrins in cell invasion and migration. *Nat Rev Cancer.* Feb 2002;2(2):91-100.

13. Beer AJ, Schwaiger M. Imaging of integrin alphavbeta3 expression. *Cancer Metastasis Rev.* Dec 2008;27(4):631-644.
14. Brower V. Tumor angiogenesis--new drugs on the block. *Nat Biotechnol.* Oct 1999;17(10):963-968.
15. Cheresh DA. Structure, function and biological properties of integrin alpha v beta 3 on human melanoma cells. *Cancer Metastasis Rev.* May 1991;10(1):3-10.
16. Haubner R. Alphavbeta3-integrin imaging: a new approach to characterise angiogenesis? *Eur J Nucl Med Mol Imaging.* Jul 2006;33 Suppl 1:54-63.
17. Kumar CC. Integrin alpha v beta 3 as a therapeutic target for blocking tumor-induced angiogenesis. *Curr Drug Targets.* Feb 2003;4(2):123-131.
18. Petitclerc E, Stromblad S, von Schalscha TL, et al. Integrin alpha(v)beta3 promotes M21 melanoma growth in human skin by regulating tumor cell survival. *Cancer Res.* Jun 1 1999;59(11):2724-2730.
19. Ruoslahti E, Pierschbacher MD. New perspectives in cell adhesion: RGD and integrins. *Science.* Oct 23 1987;238(4826):491-497.
20. Smith JW, Cheresh DA. Integrin (alpha v beta 3)-ligand interaction. Identification of a heterodimeric RGD binding site on the vitronectin receptor. *J Biol Chem.* Feb 5 1990;265(4):2168-2172.
21. Cai W, Rao J, Gambhir SS, Chen X. How molecular imaging is speeding up antiangiogenic drug development. *Mol Cancer Ther.* Nov 2006;5(11):2624-2633.
22. Cai W, Sam Gambhir S, Chen X. Multimodality tumor imaging targeting integrin alphavbeta3. *Biotechniques.* Dec 2005;39(6 Suppl):S14-25.
23. Liu S. Radiolabeled multimeric cyclic RGD peptides as integrin alphavbeta3 targeted radiotracers for tumor imaging. *Mol Pharm.* Sep-Oct 2006;3(5):472-487.
24. Cai W, Chen X. Multimodality molecular imaging of tumor angiogenesis. *J Nucl Med.* Jun 2008;49 Suppl 2:113S-128S.
25. Keereweer S, Mieog JS, Mol IM, et al. Detection of oral squamous cell carcinoma and cervical lymph node metastasis using activatable near-infrared fluorescence agents. *Arch Otolaryngol Head Neck Surg.* Jun 2011;137(6):609-615.
26. Gleysteen JP, Duncan RD, Magnuson JS, Skipper JB, Zinn K, Rosenthal EL. Fluorescently labeled cetuximab to evaluate head and neck cancer response to treatment. *Cancer Biol Ther.* Aug 2007;6(8):1181-1185.
27. Lee SB, Hassan M, Fisher R, et al. Affibody molecules for in vivo characterization of HER2-positive tumors by near-infrared imaging. *Clin Cancer Res.* Jun 15 2008;14(12):3840-3849.
28. Withrow KP, Newman JR, Skipper JB, et al. Assessment of bevacizumab conjugated to Cy5.5 for detection of head and neck cancer xenografts. *Technol Cancer Res Treat.* Feb 2008;7(1):61-66.
29. Koyama Y, Talanov VS, Bernardo M, et al. A dendrimer-based nanosized contrast agent dual-labeled for magnetic resonance and optical fluorescence imaging to localize the sentinel lymph node in mice. *J Magn Reson Imaging.* Apr 2007;25(4):866-871.
30. Kobayashi H, Kawamoto S, Bernardo M, Brechbiel MW, Knopp MV, Choyke PL. Delivery of gadolinium-labeled nanoparticles to the sentinel lymph node: comparison of the sentinel node visualization and estimations of intra-nodal gadolinium concentration by the magnetic resonance imaging. *J Control Release.* Apr 10 2006;111(3):343-351.
31. Lucarelli RT, Ogawa M, Kosaka N, Turkbey B, Kobayashi H, Choyke PL. New approaches to lymphatic imaging. *Lymphat Res Biol.* Dec 2009;7(4):205-214.
32. Jain R, Dandekar P, Patravale V. Diagnostic nanocarriers for sentinel lymph node imaging. *J Control Release.* Sep 1 2009;138(2):90-102.
33. Wallace AM, Hoh CK, Vera DR, Darrah DD, Schulteis G. Lymphoseek: a molecular radiopharmaceutical for sentinel node detection. *Ann Surg Oncol.* Jun 2003;10(5):531-538.
34. Hama Y, Koyama Y, Urano Y, Choyke PL, Kobayashi H. Two-color lymphatic mapping using Ig-conjugated near infrared optical probes. *J Invest Dermatol.* Oct 2007;127(10):2351-2356.

35. Povoski SP, Hall NC, Murrey DA, Jr., et al. Multimodal imaging and detection strategy with 124 I-labeled chimeric monoclonal antibody cG250 for accurate localization and confirmation of extent of disease during laparoscopic and open surgical resection of clear cell renal cell carcinoma. *Surg Innov.* Feb 2013;20(1):59-69.
36. Jain RK, Stylianopoulos T. Delivering nanomedicine to solid tumors. *Nat Rev Clin Oncol.* Nov 2010;7(11):653-664.
37. Keereweer S, Kerrebijn JD, van Driel PB, et al. Optical image-guided surgery--where do we stand? *Mol Imaging Biol.* Apr 2011;13(2):199-207.
38. Sampath L, Wang W, Sevick-Muraca EM. Near infrared fluorescent optical imaging for nodal staging. *J Biomed Opt.* Jul-Aug 2008;13(4):041312.
39. Schroeder A, Heller DA, Winslow MM, et al. Treating metastatic cancer with nanotechnology. *Nat Rev Cancer.* Jan 2012;12(1):39-50.
40. Yudd AP, Kempf JS, Goydos JS, Stahl TJ, Feinstein RS. Use of sentinel node lymphoscintigraphy in malignant melanoma. *Radiographics.* Mar-Apr 1999;19(2):343-353; discussion 354-346.
41. Ravizzini G, Turkbey B, Barrett T, Kobayashi H, Choyke PL. Nanoparticles in sentinel lymph node mapping. *Wiley Interdiscip Rev Nanomed Nanobiotechnol.* Nov-Dec 2009;1(6):610-623.
42. Khullar O, Frangioni JV, Grinstaff M, Colson YL. Image-guided sentinel lymph node mapping and nanotechnology-based nodal treatment in lung cancer using invisible near-infrared fluorescent light. *Semin Thorac Cardiovasc Surg.* Winter 2009;21(4):309-315.
43. Madru R, Kjellman P, Olsson F, et al. 99mTc-labeled superparamagnetic iron oxide nanoparticles for multimodality SPECT/MRI of sentinel lymph nodes. *J Nucl Med.* Mar 2012;53(3):459-463.
44. Olson ES, Jiang T, Aguilera TA, et al. Activatable cell penetrating peptides linked to nanoparticles as dual probes for in vivo fluorescence and MR imaging of proteases. *Proc Natl Acad Sci U S A.* Mar 2 2010;107(9):4311-4316.
45. Winter PM, Morawski AM, Caruthers SD, et al. Molecular imaging of angiogenesis in early-stage atherosclerosis with alpha(v)beta3-integrin-targeted nanoparticles. *Circulation.* Nov 4 2003;108(18):2270-2274.
46. Hood JD, Bednarski M, Frausto R, et al. Tumor regression by targeted gene delivery to the neovasculature. *Science.* Jun 28 2002;296(5577):2404-2407.
47. Hallahan D, Geng L, Qu S, et al. Integrin-mediated targeting of drug delivery to irradiated tumor blood vessels. *Cancer Cell.* Jan 2003;3(1):63-74.
48. Anderson SA, Rader RK, Westlin WF, et al. Magnetic resonance contrast enhancement of neovasculature with alpha(v)beta(3)-targeted nanoparticles. *Magn Reson Med.* Sep 2000;44(3):433-439.
49. Arap W, Pasqualini R, Ruoslahti E. Cancer treatment by targeted drug delivery to tumor vasculature in a mouse model. *Science.* Jan 16 1998;279(5349):377-380.
50. Yu X, Song SK, Chen J, et al. High-resolution MRI characterization of human thrombus using a novel fibrin-targeted paramagnetic nanoparticle contrast agent. *Magn Reson Med.* Dec 2000;44(6):867-872.
51. Schmieder AH, Winter PM, Caruthers SD, et al. Molecular MR imaging of melanoma angiogenesis with alphanubeta3-targeted paramagnetic nanoparticles. *Magn Reson Med.* Mar 2005;53(3):621-627.
52. Larson DR, Ow H, Vishwasrao HD, Heikal AA, Wiesner U, Webb WW. Silica Nanoparticle Architecture Determines Radiative Properties of Encapsulated Fluorophores. *Chemistry of Materials.* 2008;20(8):2677-2684.
53. Bogush GH, Tracy MA, Zukoski Iv CF. Preparation of monodisperse silica particles: Control of size and mass fraction. *Journal of Non-Crystalline Solids.* 1988;104(1):95-106.
54. Sadasivan S, Dubey AK, Li Y, Rasmussen DH. Alcoholic Solvent Effect on Silica Synthesis—NMR and DLS Investigation. *Journal of Sol-Gel Science and Technology.* 1998;12(1):5-14.

55. Herz E, Burns A, Bonner D, Wiesner U. Large Stokes-Shift Fluorescent Silica Nanoparticles with Enhanced Emission Over Free Dye for Single Excitation Multiplexing. *Macromolecular Rapid Communications.* 2009;30(22):1907-1910.

20.0 APPENDICES

Appendix 1 – Serial whole body distributions and pharmacokinetics of ^{124}I-cRGDY-PEG-Cy5-C dots

Appendix 2 – Human dosimetry of systemically administered ^{124}I-cRGDY-PEG-Cy5-C dots

Appendix 3 – Image-guided SLN mapping in a spontaneous melanoma miniswine model using cRGDY-PEG-Cy5.5- C dots

Appendix 4 – Dose escalation procedure for cRGDY-PEG-Cy5.5-C dots)

Appendix 1.

Serial whole body distributions and pharmacokinetics of ^{124}I-cRGDY-PEG-Cy5-C dots.

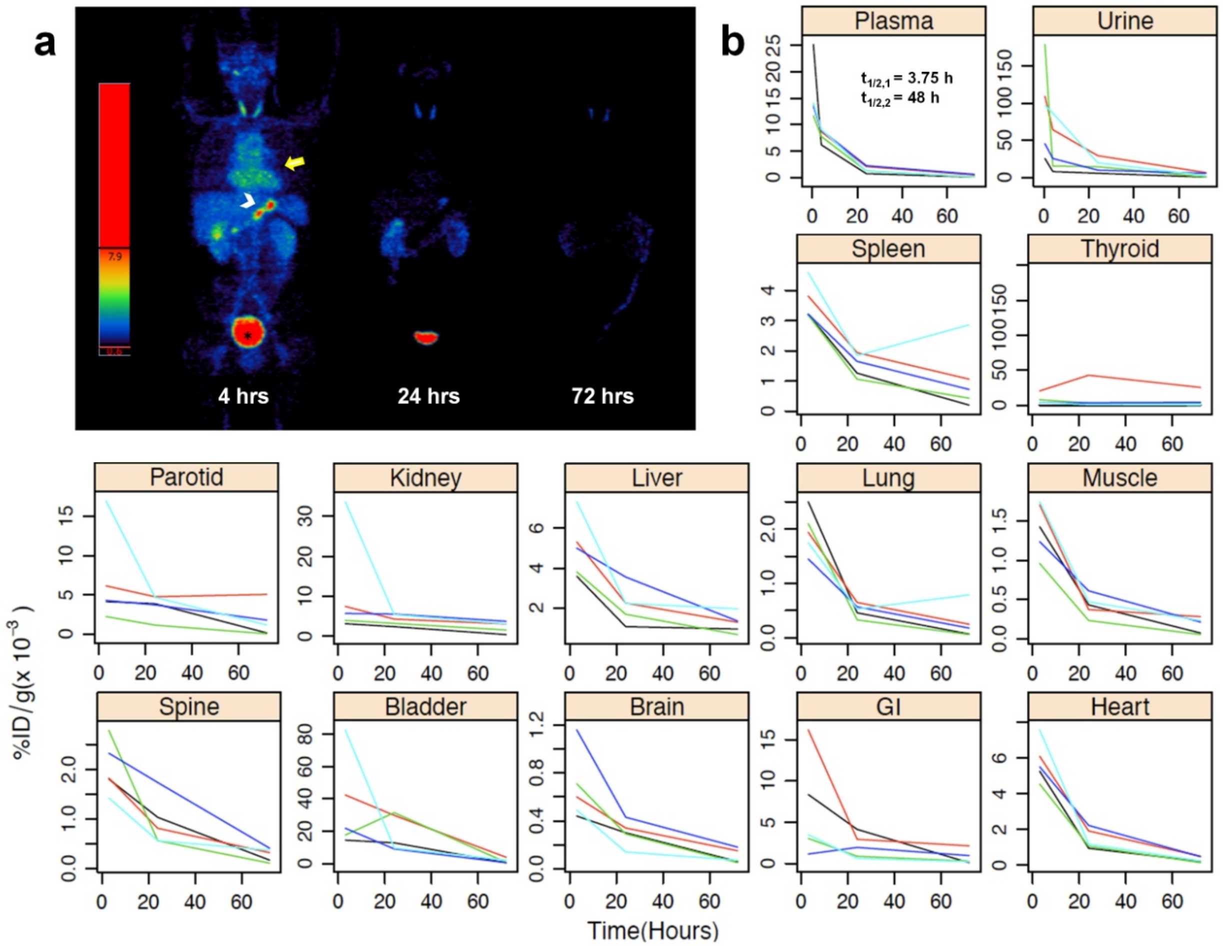

(a) Maximum intensity projection PET images at 4- (left), 24- (middle) and 72- (right) hrs p.i. of 124I-cRGDY-PEG-Cy5-C dots reveal activity in bladder (*), heart (yellow arrow), and bowel (white arrowhead). (b) Percent injected dose per gram (%ID/g) of urine and plasma collected at approximately 0.5-, 4-, 24-, and 72-hrs following injection of the particles was computed after gamma-counting and decay-correction. Image-derived regions of interest were made for each patient's PET scans over each of the displayed tissues/organs in order to derive standardized uptake values and %ID/g.

Appendix 2.

Human dosimetry of systemically administered ^{124}I-cRGDY-PEG-Cy5-C dots.

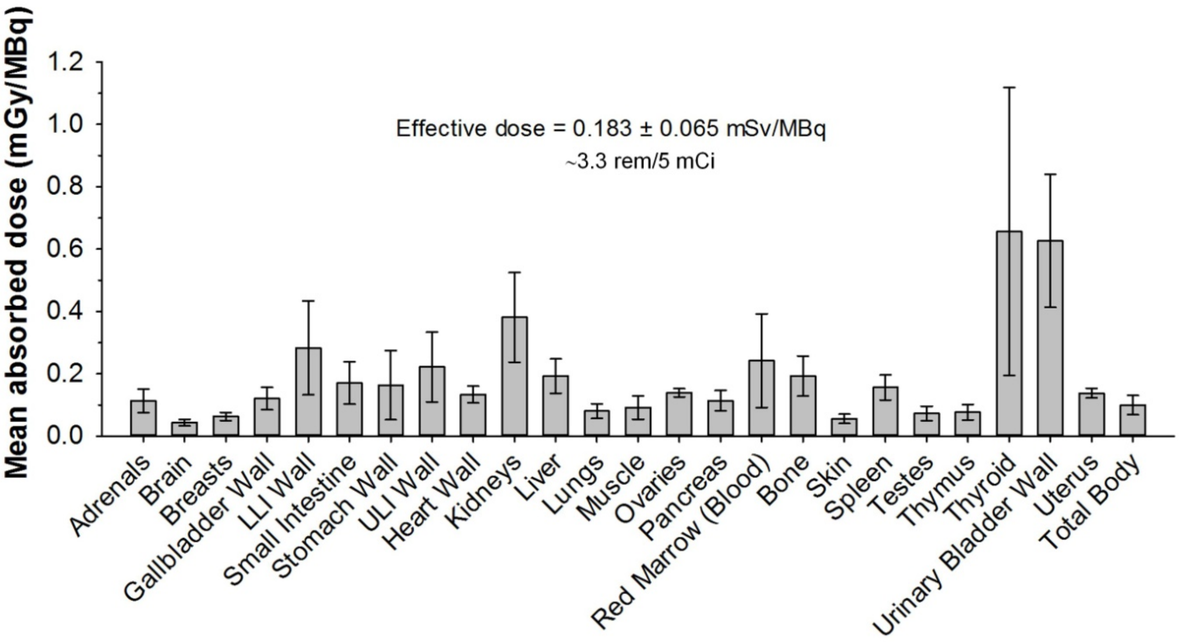

~3.3 rem/5 mCi (*versus* ^{124}I-PU-H71, 3.1 rem/mCi)

Mean normal-organ absorbed (mGy/MBq) and effective (mSv/MBq) doses of major organs and tissues (n=5) were derived from serial whole-body PET scans acquired at approximately 4-, 24-, and 72-hrs post-injection of the particle tracer using decay-corrected ROI-derived time-activity data.

Appendix 3.

Image-guided SLN mapping in a spontaneous melanoma miniswine model:
PET and real-time intraoperative optical imaging using
[^{124}]I-cRGDY-PEG-Cy5.5-C dots with correlative histology.

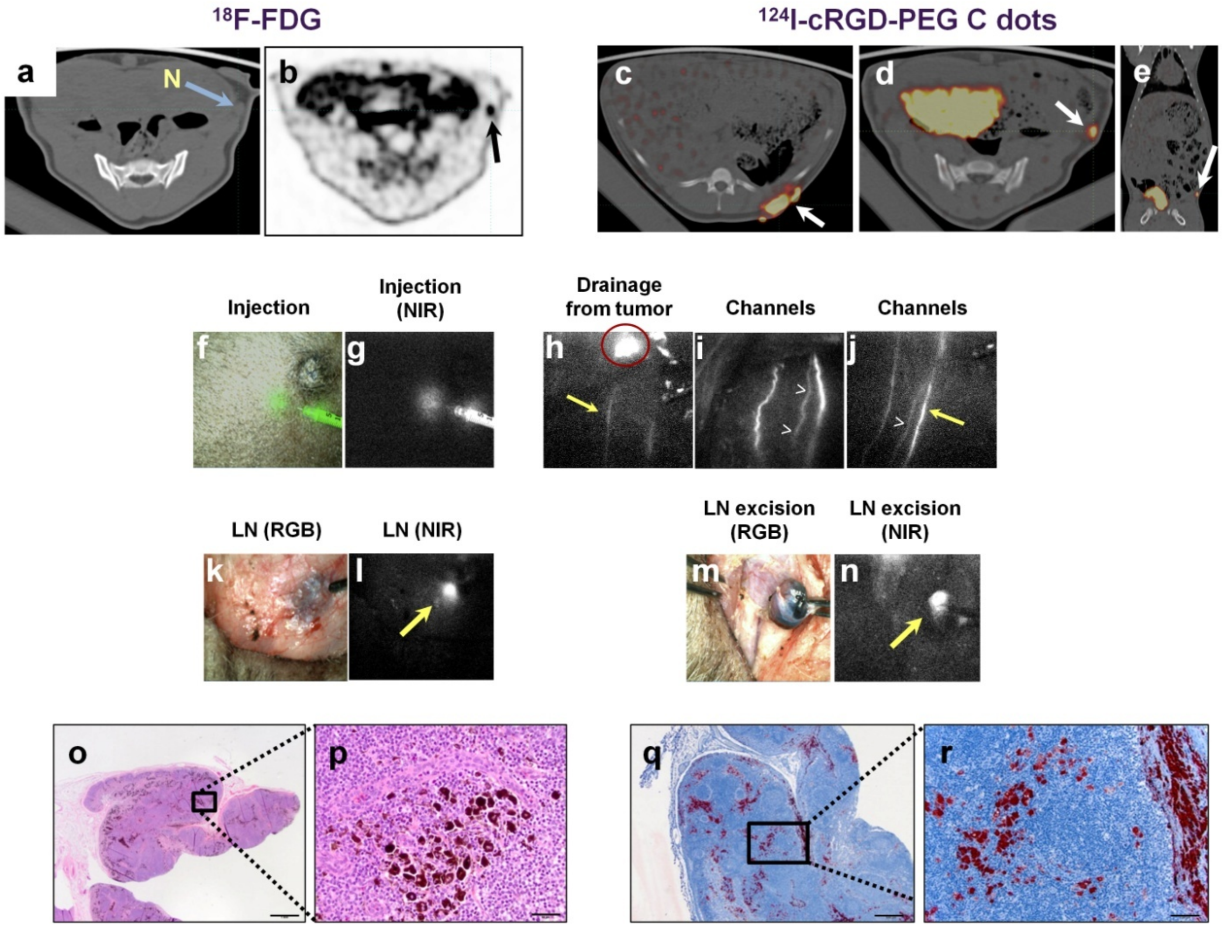

(a) Axial CT reveals a left flank SLN (arrow). (b) Axial ^{18}F-FDG PET shows localized activity within the left flank SLN (arrow) following i.v. tracer injection. (c) Axial ^{124}I-cRGDY-PEG-Cy5.5-C dot co-registered PET-CT image shows local injection site about pelvic lesion (arrow). (d) Corresponding axial and (e) coronal co-registered PET-CT images localize activity to the SLN (arrows). (f-j) Optical imaging of exposed nodal basin. Local injection of NIR dye-incorporated particles in (f) composite (green) and (g) NIR fluorescent (white) views. (h-j) Draining lymphatics distal to injection site (red circle). Fluorescence signal within main draining proximal (h,i) and mid (j) lymphatics (yellow arrows) and small caliber channels (arrowheads: i,j). SLN images: color (k,m), NIR (l,m) channels. (o) Low-power: H&E-stained SLN shows pigmented cells (bar=1 mm). (p) High-power: rounded pigmented melanoma cells and melanophages (bar=50 µm). (q) Low-power: HMB45-stained (red) SLN confirms metastases (bar=500 µm). (r) High-power: HMB45+ melanoma cell clusters (bar=100 µm).

Appendix 4.

Dose escalation procedure to establish minimum dosing in a patient (local injection cRGDY--PEG-Cy5.5-C dots) in the surgical suite

Dose range: 0.25 – 1.2 nanomoles (upper limit based on dose used for toxicity testing)
Volume: 0.1 – 0.6 ml
Concentration: 2.0 – 6.0 nmoles/ml

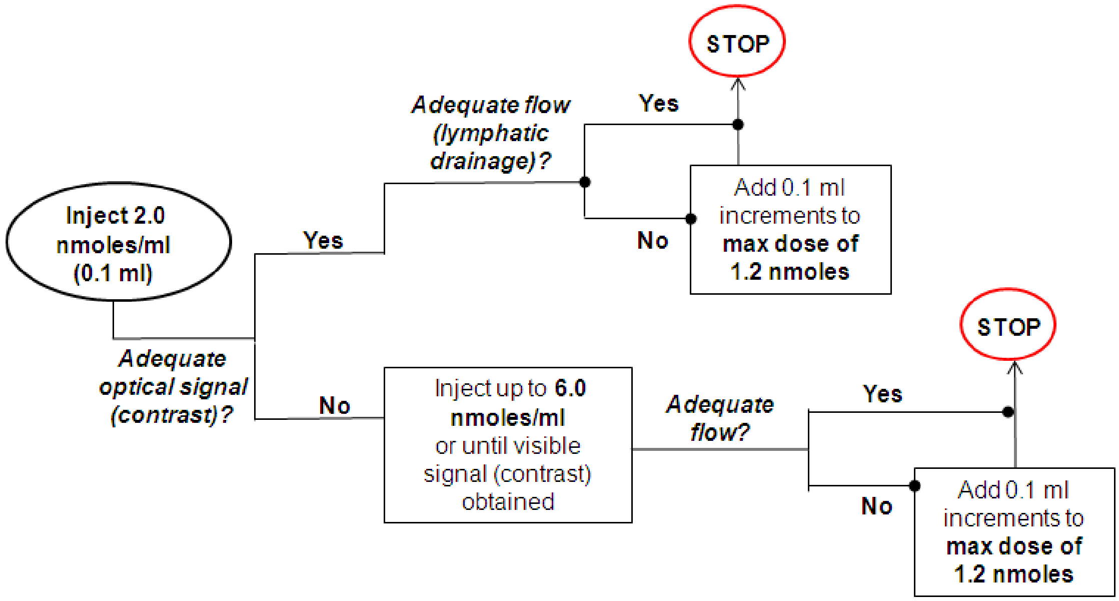

The dose escalation procedure in the surgical suite, along with the range of doses, volumes, and concentrations, is shown above. Both optical signal (image contrast) and flow to the SLN will be evaluated starting with the lowest particle concentration (2.0 nmoles/ml) and injectable volume (0.1 ml), as detailed below. If adequate signal is not observed upon injection about the primary lesion, the particle concentration will initially be increased, up to a maximum of 6.0 nanomoles/ml, until this condition is met. Upon establishing adequate optical signal (contrast), we will evaluate whether adequate flow to the SLN has occurred. If not, additional incremental volumes (0.1 ml increments) will be administered until the SLN is visualized. For any of these combinations, the maximum injectable dose is 1.2 nanomoles.

1) Several particle concentrations (minimum 2.0 nmoles/ml) will be prepared by the investigational pharmacy as 0.5-ml injectable volumes by diluting the initial base concentration (15 nanomoles/milliliter, nmoles/ml) into 0.9% sodium chloride. The maximum allowable injectable dose is 1.2 nanomoles.

2) Particle concentrations will be tested as part of a dose escalation procedure to *establish the lowest dose needed to obtain adequate fluorescence signal and detection sensitivity in humans.*

3) As particle clearance findings may in our metastatic melanoma miniswine model (or murine melanoma model) may not readily predict human tissue transport properties, we will monitor and optimize particle clearance from the site of injection to the SLN (for the optimal particle concentration) to ensure that the SLN is adequately visualized.

The above procedure will be repeated and recorded for each patient to establish optimum particle concentrations, total injection volumes, and final injected particle doses.
