# Supplementary material for: Use of Ultrasmall Core-Shell Fluorescent Silica Nanoparticles for Image-Guided Sentinel Lymph Node Biopsy in Head and Neck Melanoma: A Nonrandomized Clinical Trial
Source: JAMA Netw Open. 2021 Mar 18;4(3):e211936. doi: 10.1001/jamanetworkopen.2021.1936 (PMC7974643; doi:10.1001/jamanetworkopen.2021.1936)
Supplement: Supplement 2. — eAppendix. Supplementary Methods eFigure 1. Dose Escalation Procedure in the Surgical Suite eFigure 2. Deep Tissue SLN Detection in the Intraparotid Region eFigure 3. Fluorescence Signal of a Metastatic SLN Through Intact Skin eTable 1. Summary of QC Test Results of Representative C′ Dot Batches eTable 2. Performance of cRGDY-PEG-Cy5.5-C′ Dots for Ex Vivo SLN Identification eTable 3. Comparison of cRGDY-PEG-Cy5.5-C′ Dots to Standard of Care eReferences. [file jamanetwopen-e211936-s002.pdf]

Supplemental Online Content

Zanoni DK, Stambuk HE, Madajewski B, et al. Use of ultrasmall core-shell fluorescent silica nanoparticles for image-guided sentinel lymph node biopsy in head and neck melanoma: a nonrandomized clinical trial. *JAMA Netw Open*. 2021;4(3):e211936. doi:10.1001/jamanetworkopen.2021.1936

eAppendix. Supplementary Methods

eFigure 1. Dose Escalation Procedure in the Surgical Suite

eFigure 2. Deep Tissue SLN Detection in the Intraparotid Region

eFigure 3. Fluorescence Signal of a Metastatic SLN Through Intact Skin

eTable 1. Summary of QC Test Results of Representative C′ Dot Batches

eTable 2. Performance of cRGDY-PEG-Cy5.5-C′ Dots for Ex Vivo SLN Identification

eTable 3. Comparison of cRGDY-PEG-Cy5.5-C′ Dots to Standard of Care

eReferences.

This supplemental material has been provided by the authors to give readers additional information about their work.

© 2021 Zanoni DK et al. *JAMA Network Open.*

e Appendix. Supplementary Methods

NCCN Guidelines for Head and Neck Melanoma Treatment.

The National Comprehensive Cancer Network[1] (NCCN) guidelines for melanoma treatment in the head and neck recommend SLN biopsy for patients with clinical Stage IB or II disease. It considers SLN biopsy for patients with Stage IA disease who have adverse features (*e.g.*, very high mitotic index, particularly in the setting of young age, and/or lymphovascular invasion) or when the adequacy of microstaging is uncertain (positive deep margins ^{99m}Tc sulfur colloid.

SLN Lymphoscintigraphy and SPECT-CT

Routine pre-operative four-quadrant injection of ^{99m}Tc sulfur colloid (^{99m}TcSC; 80 MBq, 0.1 mL) about the primary melanoma site was performed on the day of surgery, followed by planar gamma camera lymphoscintigraphy and single-photon emission CT/CT (SPECT/CT) imaging of the draining lymph node basin about 0.5 – 2 hours post-injection. Once the patient was placed under general anesthesia, an experienced head and neck radiologist used anatomical landmarks and measurements from SPECT/CT images to mark the skin overlying the location of the SLN/s in each subject. These marks were used to optimize patient positioning in the operative suite and plan the surgical approach prior to conducting intraoperative fluorescence imaging and surgical excision of the primary lesion and SLNs.

Gamma Probe Detection of Radioactive SLNs Using ^{99m}TcSC

^{99m}Tc sulfur colloid radioactivity assessments were made per standard practice guidelines using a handheld intraoperative gamma probe with a collimator in place. The criteria for defining a "hot" node has been established for two decades in clinical practice and remains consistent for our current practice, including patients in this trial. Specifically, we identify SLN position based on preoperative lymphoscintigraphy, record transcutaneous SLN counts, background nodal basin count, and primary tumor count as baseline measurements prior to SLN biopsy, using the gamma probe for SLN *in vivo* assessment intraoperatively. Counts of the excised SLN are then acquired, and excision of additional nodes continues until the bed count drops to <10% of highest SLN *ex vivo* count.

Particle Synthesis and Characterization

Ultrasmall, NIR fluorescent core-shell silica nanoparticles were produced using an aqueous synthesis approach, as detailed in prior publications[2, 3] In brief, using a one-pot method, a functionalized Cy5.5 dye derivative, conjugated to a silane, was co-condensed with tetramethylorthosilicate (TMOS) to form a covalent Cy5.5 dye-encapsulating silica core in water at slightly basic pH. Particle growth was terminated by addition of α,ω-functionalized poly(ethylene glycol) (PEG), cRGDY-PEG-silane, and regular PEG-silane, producing a PEG brush shell with a fraction of the PEG chains carrying a cRGDY moiety in ω-position (i.e. on the chain end)[4].

Resulting C' dot synthesis batches (or cRGDY-PEG-Cy5.5-C'dots) were purified using gel permeation chromatography (GPC), sterile processed, and characterized by a series of quality control (QC) tests, including fluorescence correlation spectroscopy (FCS) and UV-Vis spectroscopy, as described earlier[2, 5]. QC test results summarized in **eTable 1** demonstrated high product quality and reproducibility across different production batches. On average, C' dots for clinical trials had about 1-2 Cy5.5 dyes and about 20 cRGDY integrin-targeting moieties per particle.

Multichannel Fluorescence Camera System Specifications and Real-time Optical Imaging

The imaging head of the camera system was positioned 10-30 cm above the surgical field. The overhead lights in the operating room were turned away from the operating field, and the surgical field was examined with the fluorescence camera system (irradiance < 10 mW/cm^2; limit for skin 200 mW/cm^2) to maximize detection of optical signal. Camera exposure times were less than 35 ms. Following sterile preparation of the surgical field, real-time optical scanning was performed of the particle injection site and adjacent nodal basin. Image streams were recorded from the start of injection through to biopsy and *ex vivo* imaging to document lymphatic flow and accumulation of cRGDY-PEG-Cy5.5-C' dots in SLNs. The camera was used in video mode (25-30 frames per second) to map dynamic changes in particle transit from the injection site into the draining lymphatics and nodes with successive surgical exposure. Two-dimensional large field-of-view NIR images of the adjacent soft tissue structures were also acquired for assessing the full extent of nodal groups at risk for metastasis.

© 2021 Zanoni DK et al. *JAMA Network Open.*

Signal-to-Background (SBR) Measurements of Fluorescent *In Situ* SLNs
SBR values are computed off-line, outside of the operating room setting, using a software analysis package, Architector Image Viewer (version 1.9.0), from Quest Medical Imaging, the manufacturer of the multichannel fluorescence camera system. The software is used to compute mean optical signal intensity values by selecting a specific region-of-interest (ROI) within the SLN (*in situ*) and an ROI of the surrounding tissues, the latter is used to calculate the background signal. The ROIs are subdivided into 6 regions, each region displaying a signal intensity; these signal intensity values are averaged by the software to derive mean optical signal intensities within the SLN and of the background tissues. SBRs can then be subsequently derived by dividing the mean signal intensity of the SLN by the mean signal intensity of the background. To note, SBRs cannot be calculated for *ex vivo* nodes, as there is no surrounding tissue for a background measurement.

Histopathology
Excised tissue specimens were collected, counted, and assessed optically using the same procedure as above. Specimens were histologically processed and evaluated according to standard protocols, including routine H&E staining and immunohistochemistry for melanoma biomarkers (S-100, HMB-45). Histologic results were correlated with imaging findings.

eFigure 1. Dose Escalation Procedure in the Surgical Suite

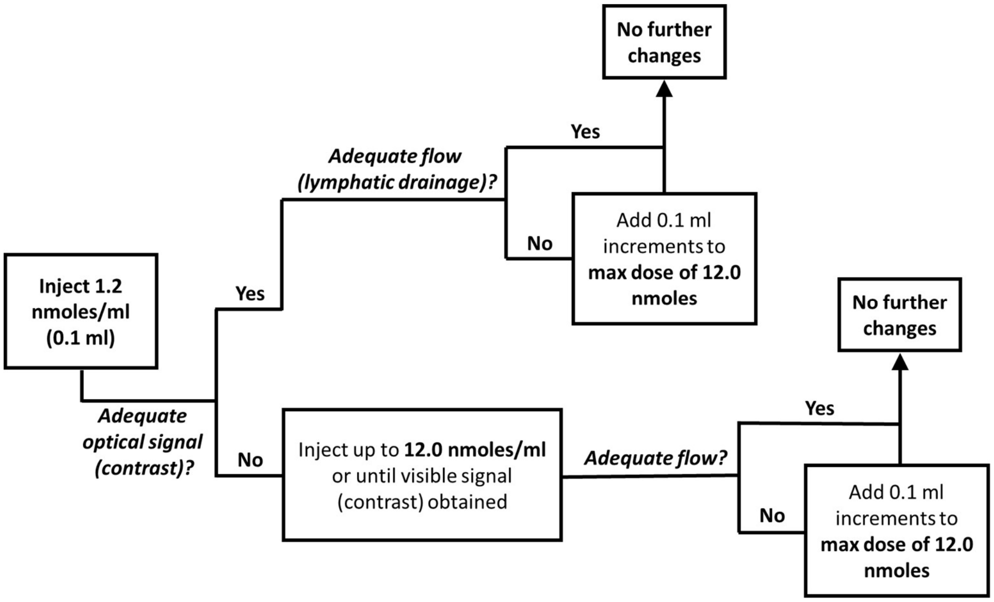

© 2021 Zanoni DK et al. *JAMA Network Open.*

eFigure 2. Deep Tissue SLN Detection in the Intraparotid Region

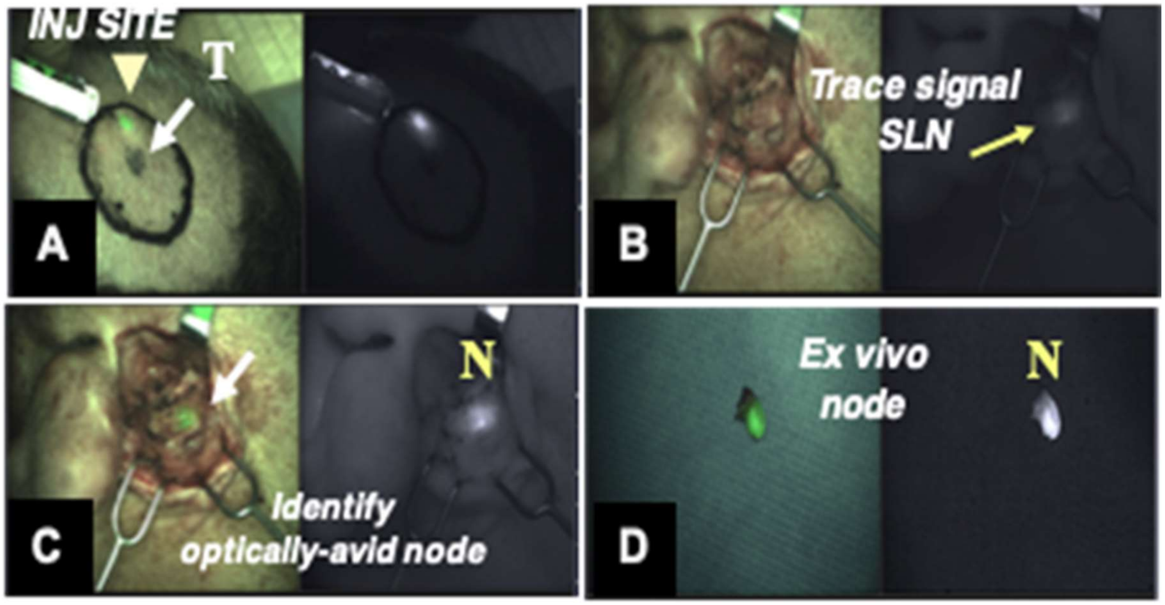

eFigure 2. 38 yr old male with a primary scalp melanoma (T) and detection of an intraparotid node 2 cm deep to the skin surface.

© 2021 Zanoni DK et al. *JAMA Network Open.*

eFigure 3. Fluorescence Signal of a Metastatic SLN Through Intact Skin

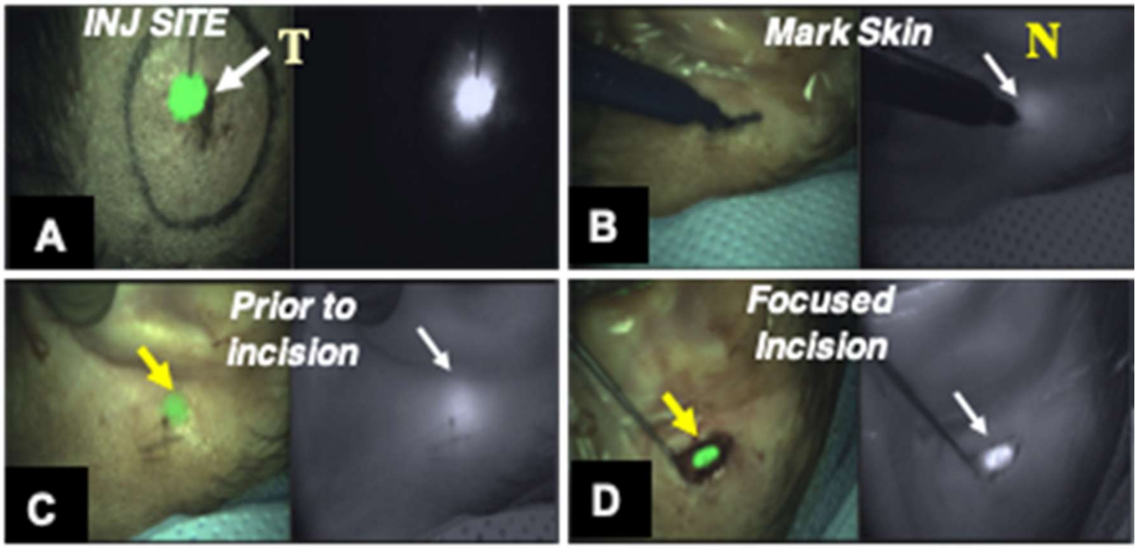

eFigure 3. 62 yr old male with a scalp melanoma (T) and fluorescence signal (arrows) overlying the site of a metastatic post-auricular SLN through the intact skin.

© 2021 Zanoni DK et al. *JAMA Network Open.*

eTable 1. Summary of QC Test Results of Representative C′ Dot Batches

Attributes (Test Methods)	Lot #1	Lot #2	Lot #3
**Purity (by GPC)**	Purity >99%	Purity >99%	Purity >99%
**Hydrodynamic diameter (by FCS)**	6.3nm	6.4nm	6.6nm
**Concentration (by FCS)**	15.0μM	15.0μM	15.0μM
**Brightness (by FCS)**	2.3 times brighter than free Cy5.5 dye	2.3 times brighter than free Cy5.5 dye	2.4 times brighter than free Cy5.5 dye
**Number of Cy5.5 per particle (by photometer and FCS)**	1.6	1.7	1.5
**Number of cRGDY per particle (by photometer and FCS)**	26	19	24

© 2021 Zanoni DK et al. *JAMA Network Open.*

eTable 2. Performance of cRGDY-PEG-Cy5.5-C′ Dots for Ex Vivo SLN Identification

Case	Phase	Dose (nanomoles)	Time from inj to nodal visualization	Pathology of resected LNs	Number of SLNs resected	Transcutaneous neck count	1st ex vivo SLN count	1st ex vivo SLN signal	1st SLN path	2nd ex vivo SLN count	2nd ex vivo SLN signal	2nd SLN path	3rd ex vivo SLN count	3rd ex vivo SLN signal	3rd SLN path	Residual bed count
1	I	0.6	~30 min	**SLN 1** negative for melanoma	1	NR	NR	yes	benign	N/A	N/A	N/A	N/A	N/A	N/A	N/A
2	I	1.2	~34 min	**R. neck SLN 1, 2, and 3** negative for melanoma	3	914	1620	yes	benign	1021	~0	benign	1466	~0	benign	~0
**3**	I	1.5	~69 min	**L. neck SLN** negative for melanoma	1	NR	1107	yes	benign	N/A	N/A	N/A	N/A	N/A	N/A	~0
**4**	I	1.2	~66 min	**R. neck SLN #1** negative for melanoma **R neck SLN #2** negative for melanoma	2	110	110	yes	benign	110	yes	benign	N/A	N/A	N/A	~0
**5**	I	2.5	~55 min	**L. retroauricular LN** negative for melanoma **L. retroauricular LN #2** metastatic melanoma	2	NR	364	yes	benign	314	yes	metastatic melanoma	N/A	N/A	N/A	148

© 2021 Zanoni DK et al. *JAMA Network Open.*

**6**	I	2.5	~40 min	**L. retroauricular SLN** negative for melanoma	1	NR	703	yes	benign	N/A	N/A	N/A	N/A	N/A	N/A	40
**7**	I	2.5	N/A	**L. ext jugular** "LN1" benign fibroadipose tissue; no LN. **L. ext jugular** "LN2"- benign fibroadipose tissue; no LN. **L. ext jugular "NS** LN"- benign fibroadipose tissue; no LN **L. occiptal LN-** intracapsular epithelioid cell LN aggregate	1	464	221	yes	benign	N/A	N/A	N//A	N/A	N/A	N/A	~0
**8**	I	0.5	~60 min	**L. neck, level 2, SLN #1**; micro-metastatic melanoma	1	2692	1119	yes	micro-metastatic melanoma	N/A	N/A	N/A	N/A	N/A	N/A	151
**9**	I	2.5	~60 min	**L. neck, SLN #1**; metastatic melanoma	1	369	2085	yes	metastatic melanoma	N/A	N/A	N/A	N/A	N/A	N/A	265

© 2021 Zanoni DK et al. *JAMA Network Open.*

**10**	I	2	~44 min	**R. neck, SLN #1** negative for melanoma **R. neck, SLN #2** negative for melanoma	2	2361	7367	yes	benign	920	yes	benign	N/A	N/A	N/A	263
**11**	I	1.5	no camera	**L. neck SLN #1** micro-metastatic melanoma **L. neck SLN #2** negative for melanoma **L. neck SLN #3** negative for melanoma **L. neck SLN #4** negative for melanoma	4	Level II 1706 Level V 606	3718	no camera	micro-metastatic melanoma	1570	no camera	benign	1930	no camera	benign	28
**12**	I	1.5	~50 min	**L. neck SLN #1** negative for melanoma **L. neck SLN #2**; lymphoid/adipose tissue,	2	NR	198	yes	benign	142	no	benign lymphoid tissue, no SLN	1245	no	benign	~0

© 2021 Zanoni DK et al. *JAMA Network Open.*

Case (Continued)	Phase (Continued)	Dose (nanomoles) (Continued)	Time from inj to first visualization (Continued)	Pathology of resected LNs (Continued)	Number of SLNs resected (Continued)	Transcutaneous neck count (Continued)	1st ex vivo SLN count (Continued)	1st ex vivo SLN signal (Continued)	1st SLN pathology (Continued)	2nd ex vivo SLN count (Continued)	2nd ex vivo SLN signal (Continued)	2nd SLN pathology (Continued)	3rd ex vivo SLN count (Continued)	3rd ex vivo SLN signal (Continued)	3rd SLN pathology (Continued)	Residual bed count (Continued)
				no SLN **L. neck SLN #3** negative for melanoma												
**13**	I	2	no camera	L. "carotid" tissue; SLN and salivary gland tissue; negative for melanoma. L. "carotid" LN; One non-sentinel LN and salivary gland tissue; negative for melanoma	1	461	919	no camera	benign	no	no	N/A	N/A	N/A	N/A	~0
**14**	I	4	~54 min	**R. neck SLN #1**; negative	1		471	yes	benign	N/A	N/A	N/A	N/A	N/A	N/A	30
**15**	I	2.5	~47 min	**R. neck ext jugular LN**; negative for melanoma.	1	SLN 1: 380<br>SLN 2: 380	344	yes	benign	N/A	N/A	N/A	N/A	N/A	N/A	~0

© 2021 Zanoni DK et al. *JAMA Network Open.*

**16**	II	2	~ 81 min	**R. neck level 2 SLN #1**; Negative for melanoma **R. neck level 2, SLN #2**; negative for melanoma	2	1227	8535	yes	benign	303	no	benign	N/A	N/A	N/A	~0
**17**	II	2	~53 min	**L post-auricular SLN**; metastatic melanoma	1	2671	3982	yes	metastatic melanoma	N/A	N/A	N/A	N/A	N/A	N/A	120
**18**	II	2	~ 59 min	**Right parotid SLN**; negative for melanoma.	1	2210	2210	yes	benign	N/A	N/A	N/A	N/A	N/A	N/A	451
**19**	II	2	~ 70 min	**L. neck level 2b, SLN# 1** negative for melanoma **L. neck level Va, SLN #2** negative for melanoma	2	200	238	yes	benign	339	yes	benign	N/A	N/A	N/A	43
**20**	II	2	~ 53 min	**R. neck ext jugular SLN #1**; negative for melanoma	2	ND	116	yes	benign	no gamma counts	yes	benign	N/A	N/A	N/A	~0

© 2021 Zanoni DK et al. *JAMA Network Open.*

				**R. neck level 2 SLN #2**; negative for melanoma												
**21**	II	2	~ 26 min	**R. neck level 2 SLN #1**; negative for melanoma **R. neck level 3, SLN #2**; negative for melanoma	2	340	354	yes	benign	331	yes	benign	N/A	N/A	N/A	300
**22**	II	2	~ 46 min	**L. neck occipital SLN #1** negative for melanoma **L. neck occiptal SLN #2** negative for melanoma	2	383	569	yes	benign	1469	yes	benign	N/A	N/A	N/A	20
**23**	II	2	~ 23 min	**R. neck ext jugular SLN #1** negative for melanoma **R. neck perifacial SLN# 2** negative for	2	SLN #1: 311<br>SLN #2: ND	380	yes	benign	574	yes,<br>(3 mm node)	benign	N/A	N/A	N/A	14

© 2021 Zanoni DK et al. *JAMA Network Open.*

				melanoma												
**24**	II	2	~ 34 min	**R. ext jugular SLN #1** metastatic melanoma **R. ext jugular SLN #2** negative for melanoma	2	SLN #1: 905<br>SLN #2: 801	3154	yes	metastatic melanoma	5272	yes	benign	N/A	N/A	N/A	267

Abbreviations: SNL, sentinel lymph node; NR, not recorded; ND, not detected; R, right; L, left; ext, external; inj, injection; path, pathology

© 2021 Zanoni DK et al. *JAMA Network Open.*

eTable 3. Comparison of cRGDY-PEG-Cy5.5-C′ Dots to Standard of Care

	**Standard of care** **^{99}Tc sulfur colloid**	**cRGDY-PEG-Cy5.5-C' dots**
**Technique**	Wider exposure of surgical field and more extensive dissection to identify SLNs; increasing potential to injure adjacent vital structures, such as nerves	Direct path to deep nodes guided by optical signal (~2.0 cm)<br><br>Less exposure and dissection needed for SLN detection, allowing targeted removal of SLNs while minimizing risk to adjacent nerves
**Anesthesia time**	Longer anesthesia times due to lack of visualization	Shorter anesthesia times due to:<br>• changes in surgical approach<br>• reduced tissue dissection<br>• identification of small SLNs not otherwise detected with standard of care agents<br>• improved discrimination of the primary lesion from SLNs
**Sensitivity**	Cannot visualize SLNs directly or discriminate nodes at different depths; cannot detect very small nodes (<4mm)	Direct high contrast visualization of SLNs through the skin or up to ~2 cm deep to the skin surface. Can detect microscopic disease (< 2-3 mm)
**Resolution**	Preoperative Scan:<br>SPECT-CT: ~5-6 mm<br>(~5 x 10^8 cells[a])	Intraoperative Scan:<br>Fluorescence camera: <60 microns<br>(approx. < 1 x 10^6 cells)

a. For ^{99m}Tc sulfur colloid: Based on a ~ 5 mm diameter focus on SPECT-CT, ~5x10^8 cells can be detected assuming, to first order, a cell density of 10^9 cells/mm^3. This is at least 2 orders of magnitude worse than the approximate cell numbers detectable with the Quest Spectrum™ fluorescence camera system.

© 2021 Zanoni DK et al. *JAMA Network Open.*

eReferences.

1. Melanoma: Cutaneous, Version 1.2021, NCCN Clinical Practice Guidelines in Oncology. November 5, 2020 https://www.nccn.org/professionals/physician_gls/pdf/cutaneous_melanoma.pdf
2. Ma K, Mendoza C, Hanson M, Werner-Zwanziger U, Zwanziger J, Wiesner U. Control of Ultrasmall Sub-10 nm Ligand-Functionalized Fluorescent Core–Shell Silica Nanoparticle Growth in Water. *Chemistry of Materials*. 2015/06/09 2015;27(11):4119-4133. doi:10.1021/acs.chemmater.5b01222
3. Ma K, Zhang D, Cong Y, Wiesner U. Elucidating the Mechanism of Silica Nanoparticle PEGylation Processes Using Fluorescence Correlation Spectroscopies. *Chemistry of Materials*. 2016/03/08 2016;28(5):1537-1545. doi:10.1021/acs.chemmater.6b00030
4. Barteau KP, Ma K, Kohle FFE, et al. Quantitative Measure of the Size Dispersity in Ultrasmall Fluorescent Organic-Inorganic Hybrid Core-Shell Silica Nanoparticles by Small-angle X-ray Scattering. *Chem Mater*. Feb 12 2019;31(3):643-657. doi:10.1021/acs.chemmater.8b04369
5. Chen F, Ma K, Benezra M, et al. Cancer-Targeting Ultrasmall Silica Nanoparticles for Clinical Translation: Physicochemical Structure and Biological Property Correlations. *Chem Mater*. 2017;29(20):8766-8779. doi:10.1021/acs.chemmater.7b03033

© 2021 Zanoni DK et al. *JAMA Network Open.*
