# Supplementary material for: Use of Ultrasmall Core-Shell Fluorescent Silica Nanoparticles for Image-Guided Sentinel Lymph Node Biopsy in Head and Neck Melanoma: A Nonrandomized Clinical Trial
Source: JAMA Netw Open. 2021 Mar 18;4(3):e211936. doi: 10.1001/jamanetworkopen.2021.1936 (PMC7974643; doi:10.1001/jamanetworkopen.2021.1936)
Supplement: Supplement 3. — Data Sharing Statement [file jamanetwopen-e211936-s003.pdf]

Data Sharing Statement

Zanoni. Use of Ultrasmall Core-Shell Fluorescent Silica Nanoparticles for Image-Guided Sentinel Lymph Node Biopsy in Head and Neck Melanoma. *JAMA Netw Open*. Published March 18, 2021. doi:10.1001/jamanetworkopen.2021.1936

Data
Data available: Yes
Data types: Deidentified participant data
How to access data: bradburm@mskcc.org
When available: With publication

Supporting Documents
Document types: None

Additional Information
Who can access the data: researchers whose proposed use of the data has been approved
Types of analyses: for a specified purpose
Mechanisms of data availability: signed data access agreement
